# Gnp4/LAX2, a RAWUL protein, interferes with the OsIAA3–OsARF25 interaction to regulate grain length via the auxin signaling pathway in rice

**DOI:** 10.1093/jxb/ery256

**Published:** 2018-07-28

**Authors:** Zhanying Zhang, Jinjie Li, Zuoshun Tang, Xingming Sun, Hongliang Zhang, Jianping Yu, Guoxin Yao, Gangling Li, Haifeng Guo, Jilong Li, Huamao Wu, Hanguang Huang, Yawen Xu, Zhigang Yin, Yanhua Qi, Rongfeng Huang, Weicai Yang, Zichao Li

**Affiliations:** 1 Key Laboratory of Crop Heterosis and Utilization, the Ministry of Education/Key Laboratory of Crop Genetic Improvement, Beijing Municipality/ College of Agronomy and Biotechnology, China Agricultural University, Beijing, China; 2 State Key Laboratory of Molecular and Developmental Biology, Institute of Genetics and Developmental Biology, Chinese Academy of Sciences, Beijing, China; 3 State Key Laboratory of Plant Physiology and Biochemistry, College of Life Sciences, Zhejiang University, Hangzhou, China; 4 Biotechnology Research Institute, Chinese Academy of Agricultural Sciences, Beijing, China

**Keywords:** Auxin, Gnp4/LAX2, grain length, rice, transcription regulation

## Abstract

Grain length is one of the determinants of yield in rice and auxin plays an important role in regulating it by mediating cell growth. Although several genes in the auxin pathway are involved in regulating grain length, the underlying molecular mechanisms remain unclear. In this study we identify a *R*ING-finger *a*nd *w*d40-*a*ssociated *u*biquitin-*l*ike (RAWUL) domain-containing protein, Gnp4/LAX2, with a hitherto unknown role in regulation of grain length by its influence on cell expansion. *Gnp4/LAX2* is broadly expressed in the plant and subcellular localization analysis shows that it encodes a nuclear protein. Overexpression of *Gnp4/LAX2* can significantly increase grain length and thousand-kernel weight. Moreover, Gnp4/LAX2 physically interacts with OsIAA3 and consequently interferes with the OsIAA3–OsARF25 interaction *in vitro* and *in vivo*. *OsIAA3* RNAi plants consistently exhibit longer grains, while the mutant *osarf25* has small grains. In addition, OsARF25 binds to the promoter of *OsERF142/SMOS1*, a regulator of organ size, and positively regulates its expression. Taken together, the results reveal that Gnp4/LAX2 functions as a regulator of grain length through participation in the OsIAA3–OsARF25–OsERF142 pathway and that it has potential value for molecular breeding in rice.

## Teaser

Gnp4/LAX2 interferes with the OsIAA3–OsARF25 interaction to activate the expression of *OsERF142/SMOS1*, and hence regulates grain length in rice.

## Introduction

Grain weight, grain number per panicle, and panicle number are major factors influencing yield in rice (*Oryza sativa*). Among them, grain weight is least affected by environmental factors (Sakamoto and Matsuoka, 2008). Nevertheless, grain weight remains a complex quantitative trait that is affected by multiple genes. Grain length, grain width, grain thickness, and grain filling rate are contributory factors that determine grain weight (Xing and Zhang, 2010). Molecular studies of each of these characters are essential for a complete understanding of their potential roles in yield improvement.

To date, many genes contributing to grain weight in rice have been isolated, such as *GS3, GW5*, *GS5*, *GL3.1*, *GW7/GL7*, *GW8, GLW7, OsGRF4*, *Big Grain1, XIAO*, *SLG*, *OsLG3*, and *OsLG3b* (Weng *et al.*, 2008; Mao *et al.*, 2010; Li *et al.*, 2011*b*; Jiang *et al.*, 2012; Wang *et al.*, 2012, 2015; Zhang *et al.*, 2012; Duan *et al.*, 2015; Liu *et al.*, 2015; Feng *et al.*, 2016; Si *et al.*, 2016; Yu *et al.*, 2017, 2018). While they all ultimately affect cell expansion or proliferation, they can be classified into several groups according to the pathways involved, including the G-protein signaling pathway, the proteasomal degradation pathway, the transcriptional regulation-related pathway, and the plant hormone biosynthesis or signaling transduction pathways (Zuo and Li, 2014; Li and Li, 2016). Although these genes have been cloned and functionally characterized, knowledge of the underlying molecular mechanisms and genetic interaction networks remain elusive and fragmentary. Consequently, it is important to isolate novel grain size-associated regulators in order to understand the molecular mechanism behind grain weight in rice.

The *R*ING-finger *a*nd *w*d40-*a*ssociated *u*biquitin-*l*ike (RAWUL) domain is a new member of the ubiquitin superfamily and has been found in the same polypeptide chain as a the RING finger domain in the polycomb repressive complex 1 (PRC1) RING family, and in the same polypeptide domain as the WDR48-p80 protein family (Sanchez-Pulido *et al.*, 2008). In *Arabidopsis*, two families of RING-finger proteins have been characterized as RAWUL domain-containing proteins, namely AtRING1A/B and AtBMI1A/B/C (Xiao and Wagner, 2015), and play roles in developmental phase transitions, cell proliferation during organ growth, and water-stress responses. However, little is known about RAWUL domain-containing proteins in rice and other crop plants.

Indole-3-acetic acid (auxin) plays an important role in growth and development of plants by regulating many biological processes (Gallavotti, 2013; Ljung, 2013). The molecular mechanisms of auxin perception are relatively well understood in different plant species (Salehin *et al.*, 2015). The auxin signaling transduction pathway consists of four components, namely the auxin receptors, the AUX/IAA repressors, the auxin response factors (ARFs), and the downstream target genes (Salehin *et al.*, 2015). Signaling transduction is initiated by the perception of auxin by the TIR1/AFB receptors that encode F-box proteins and are components of an E3 SCF ubiquitin ligase complex (Dharmasiri *et al.*, 2005). AUX/IAA co-receptors function as repressors of the pathway by directly binding to the ARF transcription factors, and together with the co-repressor protein TOPLESS they repress their activities (Szemenyei *et al.*, 2008). Auxin triggers the formation of the AUX/IAA-SCF^TIR1/AFB^ co-receptor complex and the degradation of the AUX/IAA protein in a 26S proteasome-dependent manner, which in turns results in the de-repression of the ARFs and thus the transcriptional activation of their target genes (Salehin *et al.*, 2015; Dezfulian *et al.*, 2016).

There are 31 AUX/IAAs and 25 ARF protein genes in the rice genome (Jain *et al.*, 2006; Wang *et al.*, 2007). To date, several rice AUX/IAAs have been associated with specific phenotypic effects, including root development, plant architecture, and biotic and abiotic stress responses. *OsIAA3* (referred to as *OsIAA31* by Jain *et al.*, 2006) was the first functionally characterized AUX/IAAs protein in rice and its gain-of-function causes growth defects in leaf blades and crown roots (Nakamura *et al.*, 2006). Functional analysis of ARFs have been mainly based on studies of loss-of-function mutants. For example, loss of function of *OsARF16* and *OsARF12* lead to iron-deficiency responses (Qi *et al.*, 2012; Shen *et al.*, 2013), while *OsARF23*-*OsARF24* has been shown to promote cell growth and morphogenesis by regulating *RICE MORPHOLOGY DETERMINANT (RMD*) expression (Li *et al.*, 2014). These studies highlight many aspects of plant growth that are controlled by auxin-related pathways, but the underlying regulatory processes have only been identified in a few cases. Thus, it is still not clear how these AUX/IAA and ARF networks achieve target-specificity, and whether other factors or signaling proteins participate in this regulatory process.

In our previous studies, a natural mutant with defective development of lateral spikelets on the secondary panicle branches and increased grain length was characterized and the candidate gene was designated as *Gnp4* (*Grain number per-panicle 4*), which shares the *LAX2* (*LAX PANICLE2*) locus (Tabuchi *et al.*, 2011; Zhang *et al.*, 2011*b*). Here, we report that *Gnp4/LAX2* encodes a RAWUL domain-containing protein and has a hitherto unknown role in regulating grain length. Gnp4 functions as a regulator of grain length by participating in an OsAUX/IAA–OsARF25–OsERF142 pathway.

## Materials and methods

### Plant material

Seeds of *Oryza sativa* subsp. *japonica* cv. Nipponbare and transgenic lines used in this study were generated within our laboratory. All the transgenic plants used for phenotypic evaluation were more advanced than the T_2_ generation. Rice accessions used for haplotype analysis were selected from the rice mini core collection (Zhang *et al.*, 2011*a*). The T-DNA insertion mutant *osarf25* and its wild-type (Hwayoung) were provided by Dr De’an Jiang (Zhejiang University) and the OsERF3-overexpression plants were provided by Dr Rongfeng Huang (Chinese Academy of Agricultural Sciences, Beijing).

### Plasmid construction and rice transformation

To construct the overexpression plasmid *Pro35S::Gnp4*, the full coding sequence of *Gnp4* was amplified from the cDNA of Nipponbare, digested with *Asc*I and *Spe*I, and cloned into the binary vector pMDC32 (Curtis and Grossniklaus, 2003). For construction of the GUS (β-glucuronidase) plasmid, the 2-kb promoter region of *Gnp4* was amplified from the DNA of Nipponbare, digested with *Pac*I and *Asc*I, and cloned into the binary vector pMDC162 (Curtis and Grossniklaus, 2003). For construction of GFP (green fluorescent protein) plasmids, the coding region of Gnp4 was inserted into ProSuper1330::GFP vector, and OsIAA3 and OsIAA17 were amplified and digested with *SpeI* and *AscI*, and cloned into the binary vector pMDC83 (Curtis and Grossniklaus, 2003). To construct an OsIAA3-RNAi vector, a 235-bp fragment containing part of the coding sequences and the 3′-UTR region was amplified from the cDNA of Nipponbare, digested with *Sac*I and *Spe*I, and cloned into the pTCK303 vector (Wang *et al.*, 2004) to generate the forward insertion. Next, a dsRNAi fragment obtained by digestion with *BamH*I and *Kpn*I was cloned into the same vector to generate the reverse insertion.

All plasmids were introduced into *A. tumefaciens* EHA105. Rice transformation was conducted by the *Agrobacterium*-mediated method as previously described (Hiei *et al.*, 1994). A full list of primers used in this study can be found in Supplementary Dataset S5 at *JXB* online.

### Phylogenetic analysis

The amino acid sequence of Gnp4 was used to BLAST search its closest homologous proteins from other plant species against databases in Uniprot (http://www.uniprot.org/). Multiple-sequence alignment was optimized with the Megalign program in the DNASTAR software package (http://dnastar.com). A neighbor-joining tree for homologous proteins was constructed using MEGA5.0 (Tamura *et al.*, 2011).

### GUS staining

Tissues of transgenic plants containing the *ProGnp4::GUS* vector sampled at different growth stages were fixed in GUS-staining solution [50 mM Na_2_HPO4, 10 mM Na_2_EDTA, 0.5 mM K_3_Fe (CN)6, 0.5 mM K_4_Fe (CN)6, 0.1% TritionX-100, 1 mg ml^–1^ 5-bromo-4-chloro-3-indolyl β-D-glucuronic acid]. After 12 h at 37 °C, the stained tissues were dehydrated in an ethanol series of (100%, 95%, 85%, 75%) to remove the chlorophyll, and photographed using a digital camera (Nikon D900).

### Total RNA extraction and qRT-PCR analysis

Total RNA was extracted from different plant tissues using RNAiso Plus (Takara). First-strand cDNA was synthesized in 25 μl of reaction mixture containing 2 μg Dnase I-treated RNA, 200 U M-MLV reverse transcriptase (Takara), 40 U Recombinant RNase Inhibitor (Takara), and 0.1 μΜ oligodT. Quantitative RT-PCR was carried out in total volumes of 20 μl containing 10 μl SYBR EX Taq preimix (Takara), 0.4 μl Rox Reference Dye II (Takara), 0.2 mΜ gene-specific primers, and 2 μl of first-strand cDNA on an ABI 7500 real time PCR system. *OsActin1* was used as a reference.

### Subcellular localization

The *ProSuper::Gnp4-GFP*, *Pro35S::OsIAA3-GFP*, or *Pro35S::OsIAA17-GFP* plasmids were transformed into *A. tumefaciens* EHA105 and together with the p19 strain and mCherry marker were suspended and mixed in a solution containing 10 mM 2-(N-morpholino) ethanesulfonic acid, 10 mM MgCl_2_, and 150 μM acetosyringone. After storing at 28 °C for 2 h in darkness, the mixed solution was co-infiltrated into epidermal leaf cells of *Nicotiana benthamiana.* After 3 d of incubation at 25 °C, the leaves were sampled for confocal microscopy (OlympusFV1000). The GFP and mCherry markers were excited with a 488-nm and 543-nm laser, respectively. Emission spectra were collected at 500–550 nm for GFP, and 565–615 nm for the mCherry marker.

### Yeast two-hybrid assays

The full-length and truncated fragment series of *Gnp4* were amplified and recombined into a linearized pBGKT7 vector digested with *Nde*I and *EcoR*I according to the manufacturer’s manual for the Seamless Assembly Cloning Kit (CloneSmarter, C5891). pGADT7-OsIAA3 and pGADT7-OsIAA17 plasmids were extracted from the positive clone selected from the IRAT109 cDNA library constructed by the Takara company. The bait and prey were introduced into yeast strain AH109 by the polyethylene glycol-mediated method. Experimental procedures for screening interacting candidates and plasmid isolation were conducted according to the manufacturer’s user guide (Clonetech, PT3024-1).

### Bimolecular fluorescence complementation (BiFC) assays

The full-length coding sequence of *Gnp4* without a stop codon was amplified and recombined into linearized pSPYCE(M) vectors (Waadt *et al.*, 2008) to construct Gnp4-YFP^C^. Similarly, the full-length coding sequences of *OsIAA3* and *OsIAA17* were cloned into pSPYNE173 to construct OsIAA3-YFP^N^ and OsIAA17-YFP^N^, respectively. These plasmids were transformed into *A. tumefaciens* EHA105. For transient expression the strains, together with the p19 strain and mCherry ER-rk CD3-959 (Nelson *et al.*, 2007), were co-infiltrated in 5–6-week-old *N. benthamiana* leaves. Tobacco epidermal leaf cells were observed with a confocal microscope (Olympus FV1000) 3 d after infiltration.

### Co-immunoprecipitation assays

The coding sequences of *OsIAA3* and *OsIAA17* were amplified and cloned into the *ProSuper::Myc* vector to construct *ProSuper::OsIAA3-Myc* and *ProSuper::OsIAA17-Myc*, respectively. The full-length coding sequence of *Gnp4* without the stop codon was amplified and recombined into a linearized *ProSuper::Myc* vector to construct *ProSuper::Gnp4-Myc*. Similarly, *Pro35S::HF-Gnp4* was constructed by recombining the full-length *Gnp4* with the Pro35S:HF vector. Co-immunoprecipitation was conducted as described previously (Zhang *et al.*, 2017).

### Yeast three-hybrid assays

To construct the yeast three-hybrid plasmids, full-length *OsIAA3* and *OsIAA17* were amplified and recombined into the MCSI location of the pBridge vector in the *EcoR*I-*BamH*I site, resulting in BaitI. For construction of BaitII and BaitIII, the truncated fragment and full-length *Gnp4* were amplified and recombined into the MCSII location of BaitI in the *Not*I-*Bgl*II site, respectively. OsARF25-pGADT7 was used as prey. Bait and prey were co-introduced into yeast strain AH109 and incubated at 30 °C for 3–5 d, when equal optical-density yeast cells were plated out on selective medium. Qualitative evaluation was made of the interaction activity between bait and prey.

### Determination of β-galactosidase activity

β-galactosidase activity assays with minor modification were conducted as previously described (Kippert, 1995). Yeast cells were collected and re-suspended in 800 μl Z-buffer (60 mM Na_2_HPO_4_, 40 mM NaH_2_PO_4_, 10 mM KCl, 1 mM MgSO_4_, 50 mM β-mercaptoethanol, pH 7.0), and placed on ice. β-galactosidase assays were conducted after equilibration at 30 °C for 15 min; 160 µl of 4 mg ml^–1^ o-nitrophenyl-β-d-galactoside (ONPG) was added and the mixture was thoroughly vortexed before incubatation at 30 °C. The reaction was stopped by addition of 400 μl 1M Na_2_CO_3_. The OD_550_ and OD_420_ values were determined. Three replicates were performed, each with five technical replicates.

### Scanning electron microscopy

Samples were fixed in 2.5 % glutaraldehyde and vacuumized for 30 min, then stored overnight at 4 °C. The samples were subjected to dehydration in an ethanol gradient series: 50% ×2–3 times, 70%, 80%, 90%, and 95%, each for 15 min, followed by two 20 min treatments with 100% ethanol. The dehydrated samples were treated with a mixture of equal volumes of ethanol and isoamyl acetate for 30 min and with isoamyl acetate for 1–2 h. After critical-point drying they were coated with gold using ion-beam sputtering for deposition and observed using a S-3000N scanning electron microscope (Hitachi, Tokyo, Japan).

### Transient transcriptional activity assays

The effector, reporter, and internal control plasmids were transformed into rice protoplasts using the PEG-mediated method. Total proteins were extracted using lysis buffer (Promega, E4550) after incubation at 25 °C for 12–16 h. GUS and firefly luciferase (LUC) activities were assessed as previously described (Zhang *et al.*, 2017).

### Transcriptome analysis

High-quality total RNA was extracted from 10 young panicles (1 cm in length) of Gnp4-overexpression and wild-type plants. Illumina sequencing was performed using a HiSeq2000 system at the Institute of Genetics and Developmental Biology, Chinese Academy of Sciences. Gene Ontology (GO) analysis was conducted by searching the differentially expressed genes (DEGs) against the AgriGO database of *Oryza sativa* subsp. *japonica* (http://bioinfo.cau.edu.cn/agriGO/index.php). Analysis of the significantly enriched pathways was conducted using the KEGG database (http://genome.jp/kegg/). Protein domain analyses of DEGs were conducted at DAVID (https://david.ncifcrf.gov/tools.jsp). The file containing all the 2-kb promoter sequences of the rice genome was downloaded from RAP-DB (http://rapdb.dna.affrc.go.jp/) and filtered to obtain sub-files for AuxRE *cis*-element analysis using Perl script.

### Yeast one-hybrid assays

To construct the *ProOsERF142::LacZ* and *ProOsERF3::LacZ* reporters, different promoter regions were recombined into the *EcoR*I and *Xho*I sites of the pLacZi2μ vector, respectively. For construction of pB42AD-OsARF25, full-length *OsARF25* was recombined into the *EcoR*I and *Xho*I sites of the pB42AD (pJG4-5) vector. The pB42AD-OsARF25 plasmid and the reporter constructs were co-transformed into the yeast EGY48 strain. Transformants were grown on SD/–Trp–Ura drop-out plates containing X-β-gal for blue colour development to detect the interaction. The *ProFHY1::LacZ* reporter and pB42AD-FHY3 were used as positive controls (Li *et al.*, 2010).

### ChIP-qPCR assays

The young panicles of *Pro35S::OsARF25-FLAG* plants were harvested and cross-linked, and then assays were conducted using a ChIP Assay Kit (P2078, Beyotime, China) according to the manufacturer’s instructions. The enriched DNA fragments were analysed by qRT-PCR using ABI7500 system and Software v2.0.5.

### Haplotype and nucleotide diversity analysis

The data for single nucleotide polymorphisms (SNPs) used for haplotype analysis were downloaded from the rice 3K project (RFGB, http://www.rmbreeding.cn/Index/) (Zheng *et al.*, 2015). Different haplotypes were obtained using DnaSP5.10 (Librado and Rozas, 2009) and a neighbor-joining tree for haplotypes was conducted using MEGA5.0 (Tamura *et al.*, 2011). Nucleotide diversity analysis was conducted as previously described (Sun *et al.*, 2018).

### Accession numbers

Sequence data from this work can be found in the GenBank/EMBL databases under following accession numbers: *Gnp4/LAX2* (Os04g0396500, KY673700), *OsIAA3* (Os01g0231000), *OsIAA17* (Os05g0230700), *OsARF25* (Os12g0613700), *OsERF142/SMOS1* (Os05g0389000), and *OsERF3* (Os01g0797600).

## Results

### The *gnp4* mutant shows longer grain length

**Fig. 1.**
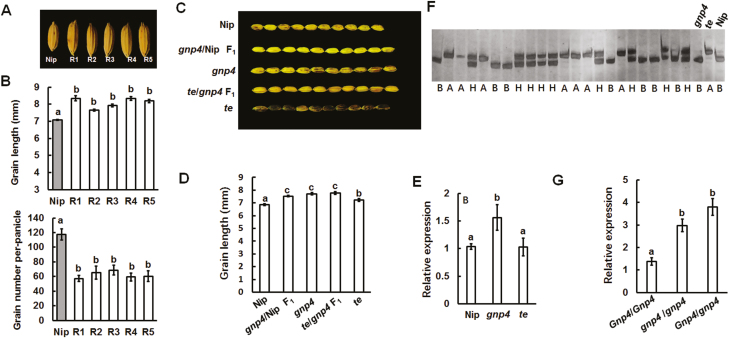
High expression of *Gnp4* correlates with longer grain length. (A) Grain morphologies of wild-type Nipponbare (Nip) and five recessive F_2_ segregates (R1–R5) of the cross between Nip and *gnp4*. (B) Grain length and grain number for Nip and the five recessive plants. Data are means (±s.e.m.) (*n*=50 grains from three panicles). (C, D) Grain morphology (C) and grain length (D) for Nip, *gnp4*, *te*, and F_1_ plants (*n*=50 grains). (E) The relative expression of *Gnp4* in Nip, *gnp4*, and *te* (*n*=4 plants). (F) Genotype analysis of *gnp4/gnp4*, *Gnp4/gnp4*, and *Gnp4/Gnp4* individuals using SDS-PAGE. (G) The relative expression of *Gnp4* in *gnp4/gnp4*, *Gnp4/gnp4*, and *Gnp4/Gnp4* individuals. Data are means (±s.e.m.) (*n*=5 plants). Different letters indicate significant differences between means according to LSD tests (*P*>0.05).

In our previous work, *Gnp4* was narrowed to a 10.7-kb region on chromosome 4, in which there was one predicted ORF (Os04g0396500). However, there was no DNA sequence difference between wild-type (WT) Nipponbare and the *gnp4* mutant except for different DNA methylation levels of several nucleotides in the promoter region (Zhang *et al*, 2011*b*). We found that the increased grain length and reduced grain number were closely linked. (Fig. 1A, B). The F_1_ seeds from the cross between *gnp4* and the WT or *gnp4* and *te*, a rice tillering mutant (Lin *et al.*, 2012), showed similar grain length to *gnp4* (Fig. 1C, D). The expression level of *Gnp4* was consistently higher in the *gnp4* mutant than the WT and *te*, and in the *gnp4/gnp4* and *Gnp4/gnp4* individuals compared with *Gnp4/Gnp4* individuals (Fig. 1E–G).

Next, we examined correlations between the expression levels of *Gnp4* and grain length and grain number per panicle in 17 *japonica* accessions randomly selected from the rice mini core collection (Zhang *et al.*, 2011*a*). We found that grain length but not grain number per panicle was correlated with *Gnp4* expression levels over 3 years (Supplementary Fig. S1A, B). These results indicated that the *gnp4* mutation was an epigenetic change that might be correlated with longer grain length with higher expression levels of *Gnp4*.

### Overexpression of *Gnp4/LAX2* increases grain length

**Fig. 2.**
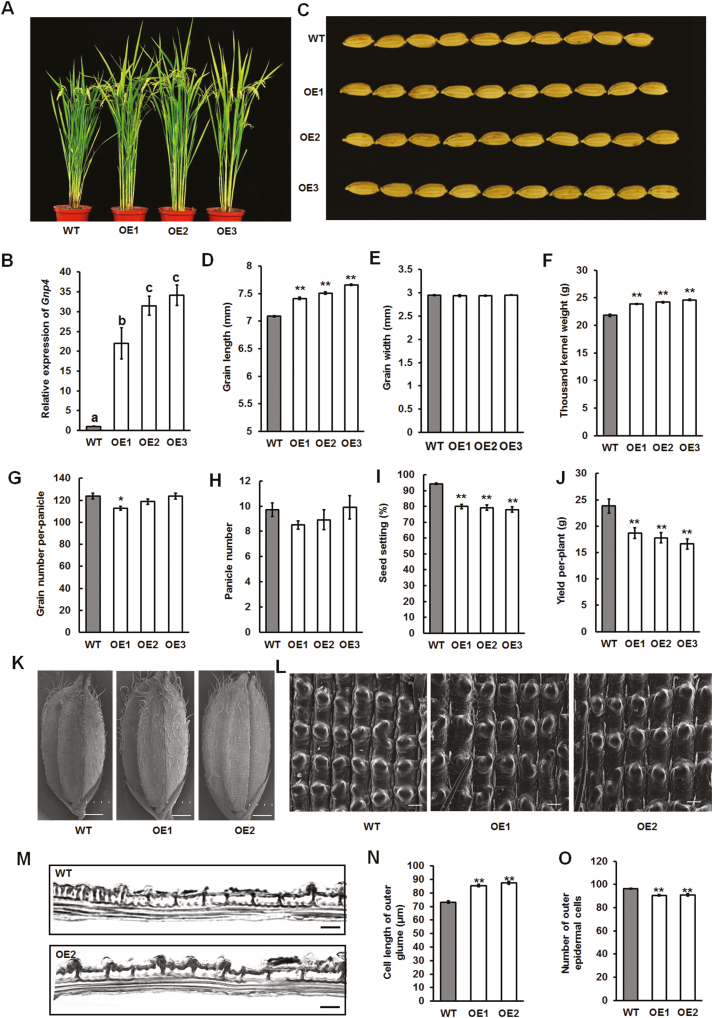
Gnp4/LAX2 is a regulator of grain length in rice. (A) Morphology of *Gnp4*-overexpression and wild-type (WT) Nipponbare plants at the mature growth stage. (B) Relative expression levels of *Gnp4* in the WT and three transgenic plants. Data are means (±s.e.m.) (*n*=3 plants, each with three technical repeats). Different letters indicate significant differences between means according to LSD tests (*P*>0.05). (C) Phenotype of grains from *Gnp4*-overexpression and WT plants. (D–J) Grain length (D), grain width (E), thousand-kernel weight (F), grain number per panicle (G), panicle number (H), seed setting (I), and yield per plant (J) of *Gnp4*-overexpression and WT plants. Data are means (±s.e.m.) (*n*=20 plants). (K) SEM of glumes of WT and *Gnp4*-overexpression plants. Scale bars are 1 mm. (L) Enlarged images of the outer surfaces of glumes. Scale bars are 50 μm. (M) Longitudinal sections of the lemma before flowering. Scale bars are 50 μm. (N) Cell length (N) and number (O) of outer glumes of *Gnp4*-overexpression and WT plants. Data are means (±s.e.m.) (*n*=12 grains). Significant differences compared with the WT were determined using Student’s *t*-test: *P<0.05, **P<0.01.

To confirm whether the expression level of *Gnp4* correlated with the grain length, an overexpression construct (*Pro35S::Gnp4*) was introduced into the Nipponbare wild-type (WT). Three overexpression lines (OE1, OE2, and OE3) showed significantly increased grain length compared to WT plants (Fig. 2A–C). The mean grain length in the lines OE1, OE2, and OE3 were about 7.41 mm, 7.51 mm, and 7.66 mm, respectively, compared to 7.09 mm in WT plants (Fig. 2D). In addition, thousand-kernel weight was increased by 9.5%, 11%, and 12.8% in the OE1, OE2, and OE3 lines, respectively, compared to WT plants, but there was little change in grain width (Fig. 2E, F). However, there was reduced yield per plant as seed setting was reduced in the *Gnp4* overexpression plants compared with the WT and there were no significant differences in grain number per panicle (except for OE1) and panicle number (Fig. 2G–J).

The spikelet glumes in rice set a limit to the final grain size, and this is determined by co-ordinated cell expansion and cell proliferation (Li and Li, 2016). We found that the mean cell length in *Gnp4* overexpression plants was significantly increased compared to the WT, but the number of epidermal cells in outer glume region decreased (Fig. 2K–O). Thus, the longer grains in *Gnp4* overexpression plants mainly resulted from enhanced cell expansion in the spikelet hulls. In addition, the expression levels of several genes conferring larger grain size by cell expansion were much higher in *Gnp4* overexpression plants than that the WT, including *GL7*, *GLW7*, *POSITIVE REGULATOR OF GRAIN LENGTH* 1 (*PGL1*), and *POSITIVE REGULATOR OF GRAIN LENGTH 2 (PGL2*) (Supplementary Fig. S2; Heang and Sassa, 2012; Wang *et al.*, 2015). Taken together, these results showed that overexpression of *Gnp4* could increase rice grain length by promotion of cell expansion.

### Subcellular localization and expression patterns of *Gnp4/LAX2*

**Fig. 3.**
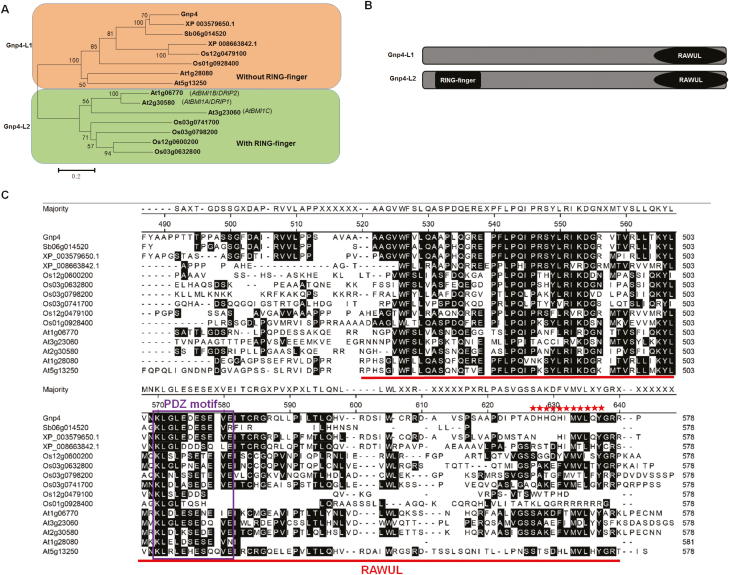
Phylogenetic tree and analysis of amino acids of Gnp4/LAX2. (A) Phylogenetic relationship among Gnp4 and homologous plant proteins. The tree was generated using the MEGA6.0 program by the neighbor-joining method. (B) Schematics of the Gnp4-L1 and Gnp4-L2 protein groups. The RING finger and RAWUL domains are indicated. (C) The amino acids of RAWUL in Gnp4 and homologous proteins. Alignment was conducted using the Megalign program in the DNASTAR software package. The RAWUL domain is indicated in red, the purple box indicates the PDZ binding motif, and the red stars indicate the amino acids required for its interaction with OsIAA3 and OsIAA17.

*Gnp4* is predicted to encode a 394-amino acid protein. We identified six Gnp4 paralogs in rice, and five orthologues in *Arabidopsis*, one in maize, one in sorghum, and one in *brachypodium distachyon* (Fig. 3A). In all cases, the proteins contained a conserved RAWUL domain at the C-terminus, and they could be classified into two groups, namely Gnp4-LIKE1 (Gnp4-L1) and Gnp4-LIKE2 (Gnp4-L2) according to the amino acid sequences at the N-termini (Fig. 3B, C). A C3H4-type zinc ring finger was present in the Gnp4-L2 group, whereas the Gnp4-L1 group was characterized by numerous stretches of the same amino acid residue, such as Arg, His, and Ser. Gnp4 belonged to the Gnp4-L1 group (Supplementary Dataset S1).

**Fig. 4.**
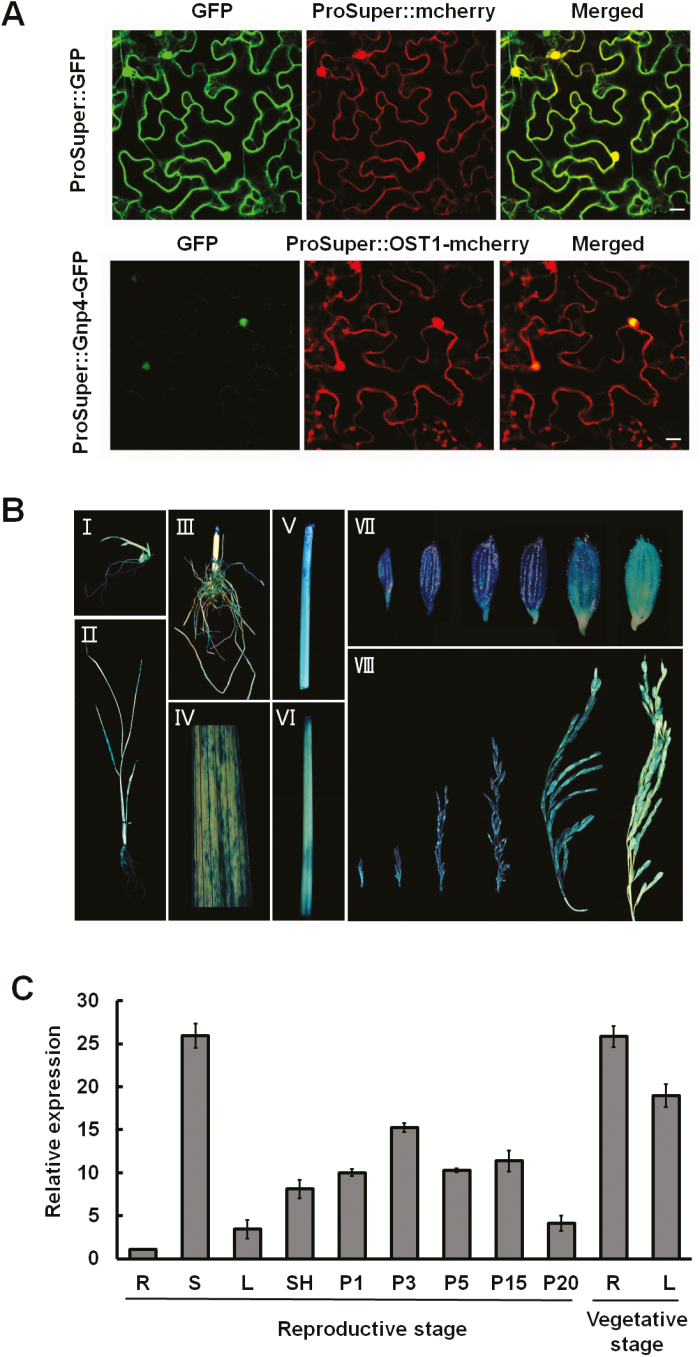
Subcellular localization and expression pattern analyses of *Gnp4/LAX2*. (A) Subcellular location of *ProSuper::GFP* and *ProSuper::Gnp4-GFP* in tobacco leaf cells. *ProSuper::OST1-mcherry* is a mCherry marker localized in the nucleus and cytosol (Ding *et al.*, 2015). Scale bars are 20 μm. (B) GUS staining of various tissues of transgenic plants containing the *ProGnp4::GUS* construct. I, bud; II, seedling; III, root; IV, leaf; V, stem; VI, sheath; VII, glumes; and VIII, panicles at different stages. (C) qRT-PCR analysis of relative expression levels of *Gnp4* in different tissues from wild-type Nipponbare. R, root; S, stem; L, leaf; SH, leaf sheath; and P, panicles at sequential lengths, where the number indicates the panicle length (cm). Data are means (±s.e.m.) (*n*=3 plants each with three technical repeats).

*Nicotiana benthamiana* leaves infiltrated with *Agrobacterium* harboring the *ProSuper::Gnp4-GFP* construct showed clear GFP signals in the nuclei, indicating that Gnp4 is a nuclear protein (Fig. 4A), which was consistent with the subcellular location of LAX2 in rice root cells (Tabuchi *et al.*, 2011). Histochemical analysis of different tissues from *ProGnp4::GUS* transgenic plants showed that *Gnp4* was widely expressed in both the vegetative and reproductive tissues, but was especially higher in stems and young panicles (Fig. 4B), consistent with previous results (Supplementary Fig. S3). RNA from roots, leaves, stems, sheaths, and panicles of different lengths were isolated and used for quantitative RT-PCR analysis of *Gnp4* expression, and the results were in agreement with the histochemical analysis (Fig. 4C). The expression pattern of *Gnp4/LAX2* was thus consistent with a role in regulating grain length.

### Gnp4/LAX2 interacts with OsIAA3 and OsIAA17 in yeast and plant cells

**Fig. 5.**
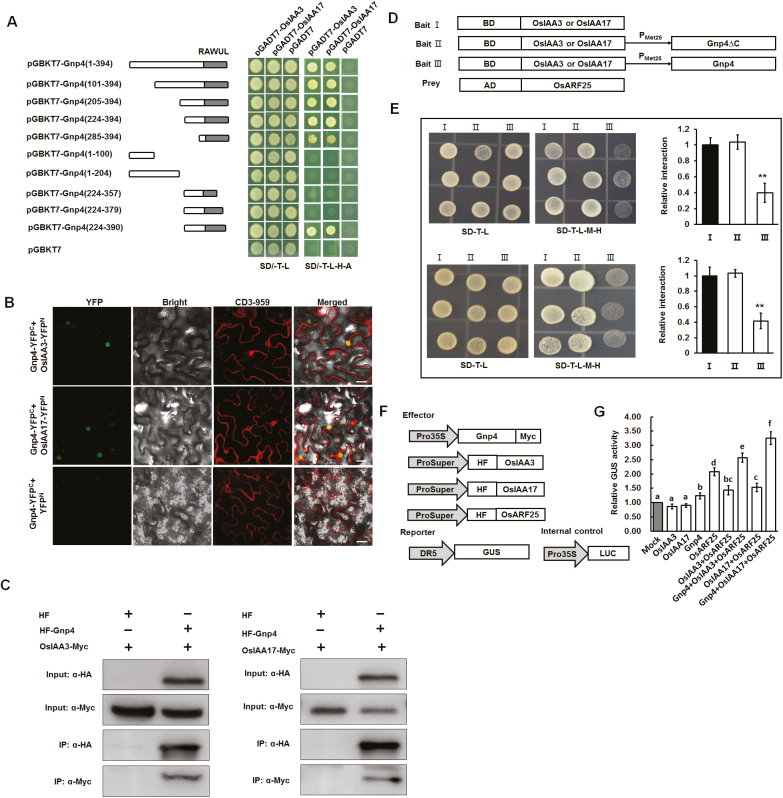
Gnp4/LAX2 interferes with the OsIAA3–OsARF25 and OsIAA17–OsARF25 interactions. (A) Gnp4 interacts with OsIAA3 and OsIAA17 in yeast cells. The RAWUL domain of Gnp4 is indicated by the gray boxes. SD/–T–L, selective medium lacking Trp and Leu; SD/–T–L–H–A, selective medium lacking Trp, Leu, His, and Ade. (B) BiFC assays showing the interactions between Gnp4 and OsIAA3 and OsIAA17 in tobacco leaf epidermal cells. CD3-959 is a mCherry marker (ER-rk CD3-959). Scale bars are 25 μm. (C) Co-immunoprecipitation assays showing that HF-Gnp4 interacts with OsIAA3-Myc and OsIAA17-Myc in plant cells. (D) Schematics of baits and prey used for yeast three-hybrid assays. (E) Yeast three-hybrid assays showing that the OsIAA3–OsARF25 (top panel) and OsIAA17–OsARF25 (bottom panel) interactions were suppressed in the presence of full-length Gnp4. Relative interaction activities were evaluated using β-galactosidase assays. Data are means (±s.e.m). Three replicates were performed, each with five technical replicates. Significant differences were determined using Student’s *t*-test: **P<0.01. (F, G) Transient assays showing the effect of Gnp4 on the OsIAA3–OsARF25 and OsIAA17–OsARF25 interactions. Relative GUS and LUC activities were measured and normalized to LUC activity. Data are means (±s.e.m.), four replicates were performed, each with five technical replicates. Different letters indicate significant differences between means according to LSD tests (*P*>0.05).

To elucidate the potential mechanism by which Gnp4 influences grain length, a yeast two-hybrid assay was performed to identify interacting proteins. Auto-transcriptional activation activity was not detected with the full-length and truncated fragment constructs of Gnp4 (Supplementary Fig. S4). Consequently, the full-length Gnp4 was used as a bait to screen two yeast prey cDNA libraries. A total of 23 candidate interacting proteins were isolated, among which OsIAA3 (*Os01g0231000*, referred to as *OsIAA3* by Jain *et al.*, 2006) and OsIAA17 (*Os05g0230700*), the nearest homologous protein of OsIAA3 in rice, were identified (Fig. 5A; Supplementary Fig. S5; Supplementary Table S1). Moreover, subcellular localization showed that OsIAA3 and OsIAA17 also localized in the nucleus and showed similar expression patterns to *Gnp4* (Supplementary Fig. S6).

Next, we found that constructs containing the RAWUL domain of Gnp4 (amino acids 101 to 394, 205 to 394, 224 to 394, 285 to 394, and 224 to 390) interacted with OsIAA3 and OsIAA17, but no interaction was observed with the constructs lacking the region corresponding to amino acids 380 to 390 (Fig. 5A). These results suggested that these 11 amino acids at the C-terminus were required for interaction between Gnp4 and OsIAA3 and OsIAA17. Furthermore, Tabuchi *et al.* (2011) identified the last 15 amino acids of LAX2 as being important for its interaction with LAX1, indicating the potentially critical function of the RAWUL domain for protein–protein interaction. However, LAX1 was not pulled down by Gnp4 in our yeast two-hybrid assays.

To further characterize the interactions between Gnp4 and OsIAA3 and OsIAA17 in plant cells, a BiFC assay was conducted by transient expression in tobacco leaf cells. As anticipated, fluorescence signals were observed in the nuclei of leaf cells where Gnp4-YFP^C^ was co-expressed with OsIAA3-YFP^N^ or OsIAA17-YFP^N^, but not with YFP^N^ alone (Fig. 5B). We also found that HF-Gnp4 proteins interacted with OsIAA3-Myc and OsIAA17-Myc proteins in tobacco leaves, but not HF proteins alone (Fig. 5C). Collectively, these results indicated that Gnp4 interacted with OsIAA3 and OsIAA17 in plant cells as well as in yeast cells.

### Gnp4/LAX2 forms a dimer and interferes with the interaction between OsAUX/IAA and OsARF

Although Gnp4 is a nuclear protein, no transcriptional activity or recognizable DNA binding domain were found, indicating that it might not regulate transcription directly. We found that Gnp4 could form dimers in yeast and plant cells (Supplementary Fig. S7). Aux/IAA proteins are well established as transcriptional repressors of the ARFs that play a key regulatory role in plant growth and development (Salehin *et al.*, 2015). Thus, we speculated that Gnp4 might function by modulating the interaction between OsAUX/IAA and OsARF. It is well documented that OsIAA3 and OsIAA17 interact with eight OsARF activators (Shen *et al.*, 2010), among which *OsARF25* showed a similar expression pattern to *Gnp4* and high expression levels during inflorescence development (Supplementary Fig. S8). Next, a yeast three-hybrid assay was performed to explore the effect of Gnp4 on the OsARF25–OsIAA3 and OsARF25–OsIAA17 interactions. We constructed three kinds of bait (Bait I, II, and III) to test for interactions with OsARF25 as prey. Bait I contained only the full-length OsIAA3 or OsIAA17, Bait II contained the C terminus-truncated Gnp4 (Gnp4∆C), and Bait III contained an entire Gnp4 (Fig. 5D). Transcription of Gnp4∆C and Gnp4 can be conditionally regulated from the Met25 promoter (ProMet25), which is actively repressed in the presence of methionine but not in its absence. We found that yeast harboring Bait III grew much more slowly than that containing Baits I or II on selective medium lacking methionine (Fig. 5E). Moreover, β-galactosidase assays showed that the OsARF25–OsIAA3 and OsARF25–OsIAA17 interactions were suppressed when Gnp4 was expressed, but not when Gnp4∆C was expressed (Fig. 5E), suggesting that Gnp4 functioned as a ‘blocker’ of the interactions, at least in yeast cells.

A transient transcriptional activity assay was then conducted to confirm that Gnp4 suppressed the OsARF25–OsIAA3 and OsARF25-OsIAA17 interactions *in planta*. We found that the relative GUS activity increased significantly when the reporter was co-expressed with *Pro35S::Gnp4-Myc* (Fig. 5F, G). Together, these results indicated that Gnp4 interfered with the OsIAA3–OsARF25 and OsIAA17–OsARF25 interactions, which may have de-repressed OsARF25 and enhanced the transcription of its downstream target genes in plant cells.

### Characterization of grain length in *OsIAA3*-RNAi and *osarf25* plants

**Fig. 6.**
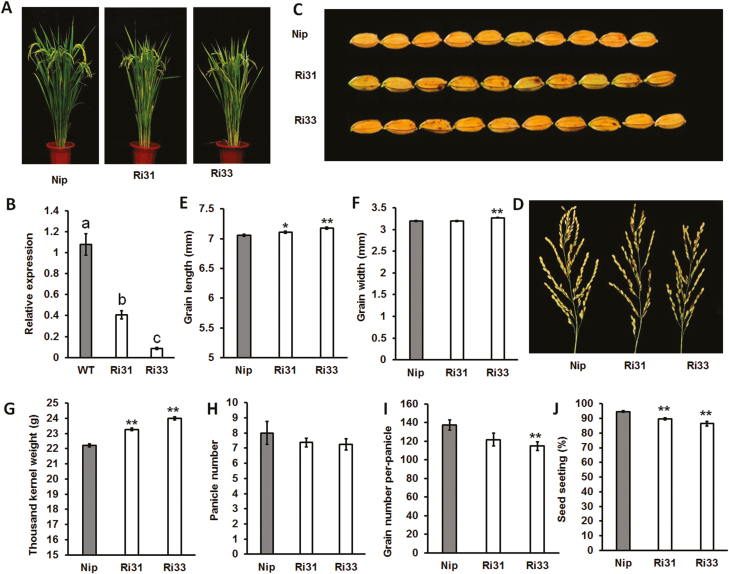
Characterization of *OsIAA3*-RNAi Plants. (A) Morphology of *OsIAA3*-RNAi plants (Ri) compared with wild-type Nipponbare (Nip) at a mature growth stage. (B) Relative expression levels of *OsIAA3* in Nip and two RNAi plants. Data are means (±s.e.m.) (*n*=3 plants, each with three technical repeats). Different letters indicate significant differences between means according to LSD tests (*P*>0.05). Phenotypes of (C) grains and (D) panicles from *OsIAA3*-RNAi and Nip plants. Grain length (E), grain width (F), thousand-kernel weight (G), panicle number (H), grain number per panicle (I), and seed setting (J) of Nip and *OsIAA3*-RNAi plants. Data are means (±s.e.m.) (*n*=15 plants). Significant differences compared with wild-type Nip were determined using Student’s *t*-test: *P<0.05, **P<0.01.

We next reasoned that the reduced expression of the *OsAUX/IAA* genes might mimic *Gnp4/LAX2* overexpression. We constructed a RNAi vector of *OsIAA3* and transformed it into the Nipponbare wild-type (WT). Two independent *OsIAA3*-RNAi plants, namely Ri31 and Ri33, were selected for detailed phenotypic analysis (Fig. 6A, B). We also checked the expression levels of several near-homologous genes of *OsIAA3* and found no significant differences between the WT and Ri31 or Ri33, except that *OsIAA19* was down-regulated in Ri31 (Supplementary Figs S5, S9). Compared with WT plants, grain length significantly increased in Ri31 and Ri33, and wider grains were observed in Ri33 (Fig. 6C–F). The thousand-kernel weights were increased by 4.7% and 8.0 % in Ri31 and Ri33 plants, respectively, compared to the WT (Fig. 6G). Similar to *Gnp4*-overexpression plants, Ri31 and Ri33 showed significantly decreased seed setting, but similar grain numbers per panicle (except for Ri33), and panicle numbers (Fig. 6H–J). These data suggested that the knockdown of *OsIAA3* expression had a positive effect on grain length.

**Fig. 7.**
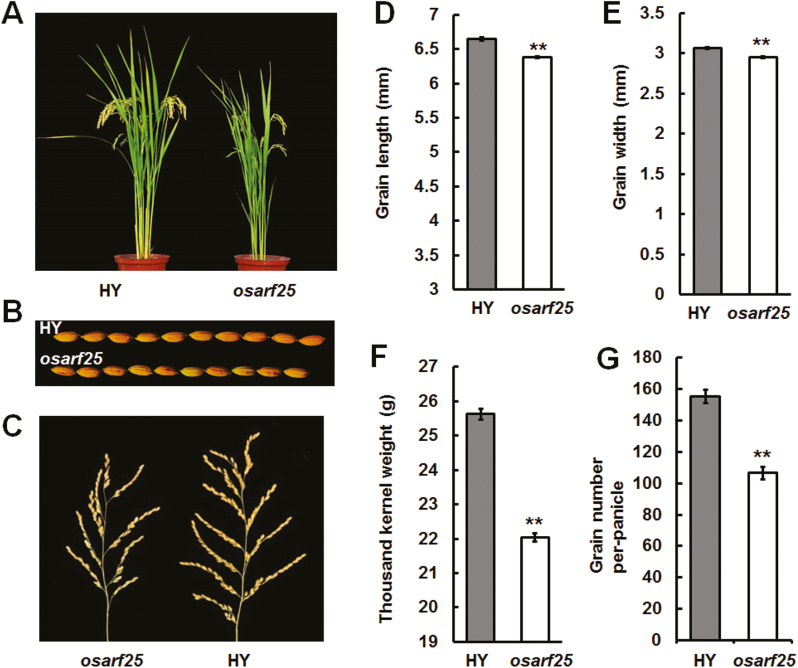
Characterization of *osarf25* plants. (A) Morphology of wild-type Hwayoung (HY) and *osarf25* plants at a mature growth stage. Phenotypes of (B) grains and (C) panicles from HY and *osarf25* plants. Grain length (D), grain width (E), thousand-kernel weight (F), and grain number per panicle (G) of *osarf25* and HY plants. Data are means (±s.e.m.) (*n*=15 plants). Significant differences compared with wild-type HY were determined using Student’s *t*-test: *P<0.05, **P<0.01.

To clarify the function of *OsARF25*, we obtained a T-DNA insertion mutant, *osarf25*, in the variety Hwayoung (HY) background. Homozygous mutant plants were identified by genomic DNA and mRNA levels (Supplementary Fig. S10). Phenotypic analysis showed that *osarf25* had smaller grains and panicles. The thousand-kernel weight and grain numbers per panicle were significantly reduced in *osarf25* relative to HY (Fig. 7). In addition, we found that the outer glume cell length, but not cell number, was significantly lower in *osarf25* than in HY (Supplementary Fig. S11). Hence, we concluded that *OsARF25* modulated grain length by cell expansion, as does *Gnp4*.

### The grain-size regulators OsERF142/SMOS1 and OsERF3 function downstream of Gnp4/LAX2 and OsARF25

To further investigate the downstream targets regulated by *Gnp4*, transcriptome analysis was performed for *Gnp4*-overexpression and wild-type plants. A total 846 and 449 genes were up- and down-regulated, respectively, in *Gnp4*-overexpression plants compared to the WT (Supplementary Dataset S2). The expression levels of several genes were quantified by qRT-PCR to confirm the RNA-seq results (Supplementary Fig. S12). Gene ontology (GO) analysis showed that genes affected by the overexpression of *Gnp4* were significantly enriched in 40 GO terms (Supplementary Table S2). Among these terms, several specific GO keywords were consistent with the molecular function of *Gnp4*, such as GO: 0045449 (regulation of transcription), GO: 0003700 (transcription factor activity), GO: 0030528 (transcription regulator activity), and GO: 0005634 (nucleus) (Supplementary Fig. S13). Two KEGG pathways were enriched, namely ko04075 (plant hormone signal transduction) and ko04626 (plant–pathogen interaction) (Supplementary Fig. S14). These results were consistent with our proposed role for *Gnp4* in the regulation of grain size.

We also analysed the gene functional categories of the up- and down-regulated genes using the DAVID database. Five terms were significantly enriched for the up-regulated genes and one term was enriched for down-regulated genes (Supplementary Table S3). Among the up-regulated genes, the most significantly enriched was the AP2 (APETALA2) domain, which plays roles in various biological processes of plant development and abiotic and biotic stress responses (Licausi *et al.*, 2013). These results provided hints on the downstream targets of *Gnp4* that are potentially involved in regulating grain length. A total of 28 genes encoding AP2 domain-containing proteins were found to be up-regulated in *Gnp4*-overexpression plants and were selected for further investigation. Interestingly, the AuxRE (TGTCTC) *cis*-element was found in the 2-kb promoter region of 18 of these genes (Supplementary Table S4). Among them, *OsERF142* (also known as *SMOS1* and *SHB*) is well known to influence organ size through the auxin signaling pathway, and to modulate root meristem size by influencing GA biosynthesis (Aya *et al.*, 2014; Li *et al.*, 2015), and *OsERF3* is known to be involved in root development, drought tolerance, and defense responses in rice (Lu *et al.*, 2011; Wan *et al.*, 2011; Zhao *et al.*, 2015). A recently isolated novel AP2 domain-containing protein, OsLG3/OsERF62, has been shown to be a positive regulator of grain length (Yu *et al.*, 2017). Next, we generated *OsERF3*-overexpression plants in the Nipponbare background and found that several independent transgenic lines showed significant increases in grain length and thousand-kernel weight compared to the WT plants, indicating that *OsERF3* was a positive regulator of grain length (Supplementary Fig. S15). We thus speculated that *OsERF142* and *OsERF3* might be common targets of OsARF25 and act downstream of Gnp4.

**Fig. 8.**
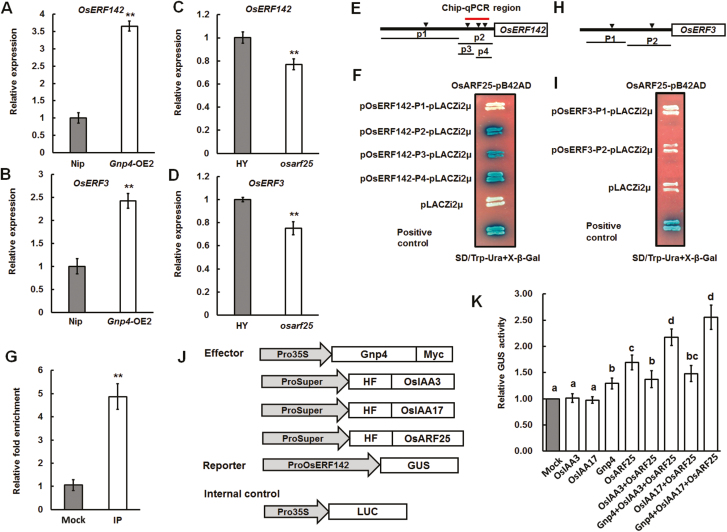
Gnp4/LAX2 regulates *OsERF142* and *OsERF3* expression. (A–D) Relative expression levels of *OsERF142* and *OsERF3* in *Gnp4*-overexpression and wild-type Nipponbare (Nip) plants or in *osarf25* and wild-type Hwayoung (HY). Data are means (±s.e.m.) (*n*=3 plants each with three technical repeats). Student’s *t*-test. **P<0.01. (E, F) *ProOsERF142* used in yeast one-hybrid assays showing the binding activity of OsARF25 to the P2 region of *ProOsERF142*. AuxRE *cis*-elements are indicated with black triangles. (G) ChIP–qPCR indicating the binding of OsARF25 to the P2 region of *ProOsERF142*. Data are means (±s.e.m.) (*n*=3). Student’s *t*-test: **P<0.01. (H, I) *ProOsERF3* used in yeast one-hybrid assays showing no interaction between OsARF25 and fragments of *ProOsERF3*. (J, K) Effectors, reporter, and internal control used in the transient assays to determine the effect of Gnp4 on transcription of *OsERF142*. Data are means (±s.e.m.). There were four replicates, each with five technical replicates. Different letters indicate significant differences between means according to LSD tests (*P*>0.05).

To test the hypothesis, we first checked the expression levels of *OsERF142* and *OsERF3*, and found that the mRNA abundance was much higher in *Gnp4*-overexpression plants than in the WT (Fig. 8A, B). In contrast, reduced expression of *OsARF2*5 led to significantly decreased expression levels of *OsERF142* and *OsERF3* in the *osarf25* mutant in the HY background (Fig. 8C, D). Next, yeast one-hybrid and CHIP-qPCR assays confirmed that OsARF25 binds to the P2 region of Pro*OsERF142*. (Fig. 8E–G). However, we could not detect any interactions between OsARF25-pB42AD and fragments of *ProOsERF3* in yeast cells (Fig. 8H, I). In addition, we found elevated GUS activity driven by the *OsERF142* promoter when Gnp4 was co-expressed compared with controls (Fig. 8J, K). These results collectively indicated that *OsERF142* and *OsERF3* function downstream of Gnp4 and OsARF25, and that OsARF25 binds to the promoter of *OsERF142*/*SMOS1* and positively regulates its expression.

### Nucleotide diversity and haplotype analysis of Gnp4/LAX2

In previous studies, several quantitative trait loci located near *Gnp4* conferring grain length have been isolated in different rice cultivars, such as *GWT4a*, *qLWR4*, and *qGL4b* (Lin *et al.*, 1996; Ying *et al.*, 2012; Segami *et al.*, 2016). To investigate the natural variation of *Gnp4* in different germplasm types, we further analysed the sequences of *Gnp4* in 259 cultivated and nine wild rice types and found 17 haplotypes (Hap) based on 46 SNPs. Hap1-Hap5 and Hap8 were mainly present in the *indica* subpopulation (sub-I), Hap6, Hap7, Hap9, and Hap10 were mainly present in the *japonica* subpopulation (sub-J), and Hap11-Hap17 were present in wild rice (Supplementary Fig. S16; Supplementary Dataset S3). This analysis showed an obvious diversification of the *Gnp4* locus in the *indica* and *japonica* subspecies. Furthermore, we found that the nucleotide diversity of *Gnp4* in *japonica* (π=0.00016) was much lower than that in *indica* (π=0.00093) and in wild rice (π=0.0021). Significant Tajima’s *D* and Fu Li’s *D* values were also observed in *japonica* (Supplementary Table S5). These results indicated that *Gnp4* alleles in the *japonica* subpopulation might have been selected during domestication.

## Discussion

### Gnp4/LAX2 functions as a subset of the auxin response pathway

**Fig. 9.**
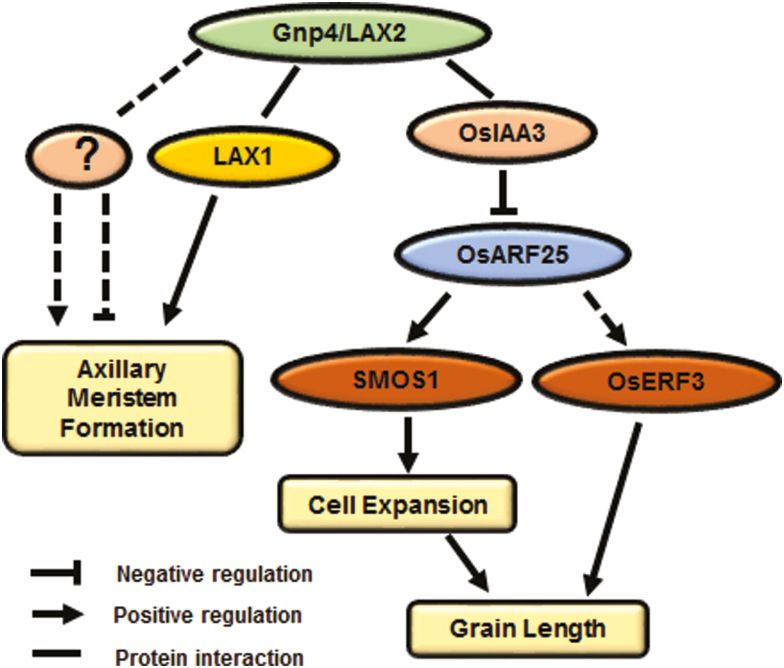
A proposed model for the functions of Gnp4/LAX2. Gnp4/LAX2 encodes a RAWUL domain-containing protein. It is involved in axillary meristem formation and acts in the LAX1-dependent and LAX1-independent pathways (Tabuchi *et al.*, 2011). In addition, Gnp4/LAX2 interacts with OsAUX/IAAs, such as OsIAA3 and OsIAA17. AUX/IAAs function in plant cells as transcription repressors of ARFs. With elevated mRNA levels, the abundance of Gnp4 possibly functions as a ‘blocker’ of the OsIAA3–OsARF25 interaction and thus enhances transcription of downstream target genes. OsARF25 acts upstream of OsERF142 and OsERF3 and directly binds to the promoter of *OsERF142*, which positively regulates cell expansion, consequently affecting phenotypic variation in grain length.

Although the biosynthesis, transportation, and signal transduction processes of auxin have been well studied, the regulators of components of the auxin pathway are largely unknown. In this study, we found a component of auxin signaling involved in the regulation of grain length in rice. Gnp4/LAX2 physically interacted with OsIAA3 and OsIAA17 in yeast and plant cells, and affected the interaction between them with OsARF25 (Fig. 5). *OsIAA3-RNAi* plants displayed a phenotype with longer grain length similar to that of *Gnp4*-overexpression plants, while the loss-of-function *osarf25* mutant had small grains (Fig. 7), leading us to propose that Gnp4 interfered with the OsIAA3–OsARF25 interaction and increased the expression of OsARF25 target genes, including *OsERF142* and *OsERF3*. The rice *smos1* (*oserf142*) mutant has small organ size due to decreased cell size (Aya *et al.*, 2014). SMOS1 (OsERF142) interacts with SMOS2 (also known as Dwarf and Low Tillering, DLT) to from a complex that regulates the expression of its direct target, *OsPHI-1*, which is involved in cell expansion (Hirano *et al.*, 2017). In addition, expression levels of *OsERF142* and *OsPHI-1* were decreased in plants overexpressing *OsIAA3* (P58L, a constitutively active form of *OsIAA31* named by Jain *et al.*, 2006) (Hirano *et al.*, 2017). We found that *OsERF142* was up-regulated in *Gnp4*-overexpression plants compared to the Nipponbare wild-type (WT), but was down-regulated in *osarf25* compared to the HY wild-type (Fig. 8A, C). In addition, up-regulation of *OsPHI-1* and three *OsPHI-1-like* genes in *Gnp4*-overexpression plants were detected in our RNA-seq data (Supplementary Dataset S24). Moreover, microscopy showed that the cell length increased significantly in *Gnp4*-overexpression plants but decreased in *osarf25*, resulting from changes in cell expansion (Fig. 2K–O; Supplementary Fig. S11). Based on our results and previous reports, we propose that Gnp4 might function in an OsIAA3–OsARF25–OsERF142 pathway to regulate grain length as shown in Fig. 9. It has been reported that LAX2 interacts with LAX1, which is involved with the auxin and brassinosteroid signal transduction pathways, to regulate the process of axillary meristem formation; moreover, LAX1 interacts with LAX2, and *LAX1*-overexpression plants also show decreased seed setting, similar to *Gnp4/LAX2*-overexpression plants (Komatsu *et al.*, 2003; Tabuchi *et al.*, 2011). So Gnp4/LAX2 may function as a subset of the auxin response pathway to regulate axillary meristems and pollen formation or grain development through different interacting protein-dependent pathways.

### Gnp4/LAX2 functions as a regulator of protein interactions

Several studies on Gnp4-L2 proteins have been carried out in *Arabidopsis*. Overexpression of *AtBMI1C* accelerates flowering by repression of the *FLOWERING LOCUS C* (*FLC*) gene (Li *et al.*, 2011*a*) and *AtRING1A/B* regulates cell-fate differentiation by suppressing expression of Class I *KNOTTED-like homeobox* (*KNOX*) genes (Xu and Shen, 2008). These proteins function as transcriptional repressors. Post-translational modification of potential targets of some of these Gnp4-L2 homologs have also been described, suggesting a possible E3 ligase activity of these ring finger domain-containing proteins. *AtBMI1A/B*, also known as *DREB2A-INTERACTING PROTEIN1* (*DRIP1*), and *DRIP2* negatively regulate the response to water stress through interaction with DREB2A and mediating its degradation (Qin *et al.*, 2008). Here, we found that Gnp4 functions as a regulator of grain size in rice, indicating that the Gnp4-L1 and Gnp4-L2 proteins may use different mechanisms to regulate plant growth. Compared to Gnp4-L2, Gnp4-L1 proteins do not have a RING finger domain at the N terminus, which is required for the commonest class of E3 ubiquitin ligases, suggesting that they lack this activity. Moreover, a PDZ (post-synaptic density protein *P*SD95, Drosophila disc large tumor suppressor *D*lg1 and tight junction protein *Z*O-1) binding motif (KLGLEDSEV) has been found in the RAWUL domain of Gnp4 (Wardell, 2013). This motif usually functions as a regulator of protein–protein interactions or dimer formation (Fanning and Anderson, 1996; Short *et al.*, 1998) (Fig.3). We also found that 368 proteins in rice contained the core sequence ‘LGLE’ of the PDZ motif, including OsIAA3 (Supplementary Dataset S4). Together, our results showed that Gnp4 forms a dimer and functions as a regulator of protein–protein interactions (Fig. 5; Supplementary Fig. S7).

### Manipulation of the expression level of *Gnp4/LAX2* has the potential to improve grain yield

Our results demonstrated that Gnp4 functions as a regulator of grain length. Overexpression of *Gnp4* significantly increased the grain length and thousand-kernel weight of rice, indicating its potential value for breeding. However, we found that seed setting was decreased in *Gnp4*-overexpression plants (Fig. 2). When we evaluated correlations of *Gnp4* expression levels with seed setting and yield per plant in 17 *japonica* accessions, we found that accessions with 7–10-fold increases in *Gnp4* expression levels relative to Nipponbare exhibited high yields, implying that there is an appropriate level of *Gnp4* expression that might be required for yield improvement (Supplementary Fig. S1). Constitutive overexpression of *CBP1* (*CLUSTERED PRIMARY BRANCH 1*) under the control of the maize ubiquitin promoter has been shown to increase grain length but not yield per plant, a result of other unfavorable agronomic traits; however, optimized expression of *CBP1* using a panicle-specific promoter did result in improved yield in rice (Wu *et al.*, 2016). In a similar way, it would be interesting to test whether controlled expression of *Gnp4* in specific tissues could be used to improve yields in rice.

## Supplementary data

Supplementary data are available at *JXB* online.

Fig. S1. Analysis of the correlation between *Gnp4/LAX2* mRNA levels and several agronomic traits in 17 *japonica* accessions.

Fig. S2. Relative expression levels of several genes related to grain length in rice.

Fig. S3. *In silico* expression analysis of *Gnp4/LAX2*.

Fig. S4. Auto-transcriptional activation activity analysis of Gnp4/LAX2 in yeast cells.

Fig. S5. Phylogenetic tree of Aux/IAA proteins in rice.

Fig. S6. Subcellular localization and expression pattern of *OsIAA3* and *OsIAA17*.

Fig. S7. Gnp4 forms a dimmer in yeast and plant cells.

Fig. S8. Expression pattern analysis of *OsARF25*.

Fig. S9. Relative expression levels of nearest homologous genes of *OsIAA3* in wild-type Nipponbare and *OsIAA3*-RNAi plants.

Fig. S10. Identification of *osarf25*.

Fig. S11. Scanning electron microscopy of glumes of wild-type Hwayoung and *osarf25*.

Fig. S12. Validation of transcriptome data by qRT-PCR.

Fig. S13. Gene ontology analysis of DEGs.

Fig. S14. KEGG pathway analysis of DEGs.

Fig. S15. Phenotypic analysis of *OsERF3*-overexpression and wild-type plants.

Fig. S16. Haplotype analysis of *Gnp4/LAX2*.

Table S1. Gnp4/LAX2-interacting proteins isolated by yeast two-hybrid assays.

Table S2. Enriched GO terms in significant DEGs.

Table S3. Significantly enriched protein domains of up- and down-regulated genes in *Gnp4/LAX2*-overexpression compared to wild-type plants.

Table S4. DGEs containing AP2 domains in *Gnp4/LAX2*-overexpression compared to wild-type plants.

Table S5. The nucleotide diversity of *Gnp4/LAX2*.

Dataset S1. Amino acid alignments of Gnp4/LAX2 and its homologous proteins.

Dataset S2. DEGs in *Gnp4/LAX2*-overexpression compared to wild-type plants.

Dataset S3. Details of *Oryza sativa* varieties and wild rice genotypes.

Dataset S4. Proteins in rice containing the core sequence ‘LGLE’ of the PDZ motif.

Dataset S5. Primers used in this study.

## Author contributions

ZZ designed and performed the research and wrote the article; Jinjie Li contributed to supervising the research and revising the manuscript; ZT contributed to the transcriptome experiment; XS contributed to helping with the transgenic experiment; HZ supervised the research; JY contributed to helping with the haplotype analysis; GY, GL, HG, Jilong Li, WH, HH, YX, and ZY contributed to the preparation of samples or reagents; YQ contributed to the identification of the *osarf25* mutant; RH contributed to the *Pro35S::OsERF3* transgenic formation; WY contributed to transcriptome experiment; and ZL conceived the research and assisted in writing the manuscript.

## Supplementary Material

Supplementary Figures S1-S16

Supplementary Tables S1-S5

Supplementary Dataset S1

Supplementary Dataset S2

Supplementary Dataset S3

Supplementary Dataset S4

Supplementary Dataset S5
