# Supplementary material for: Gnp4/LAX2, a RAWUL protein, interferes with the OsIAA3–OsARF25 interaction to regulate grain length via the auxin signaling pathway in rice
Source: J Exp Bot. 2018 Jul 28;69(20):4723–37. doi: 10.1093/jxb/ery256 (PMC6137978; doi:10.1093/jxb/ery256)
Supplement: Supplementary Figures S1-S16 [file ery256_suppl_supplementary-figures-s1-s16.pdf]

Supplementary figures

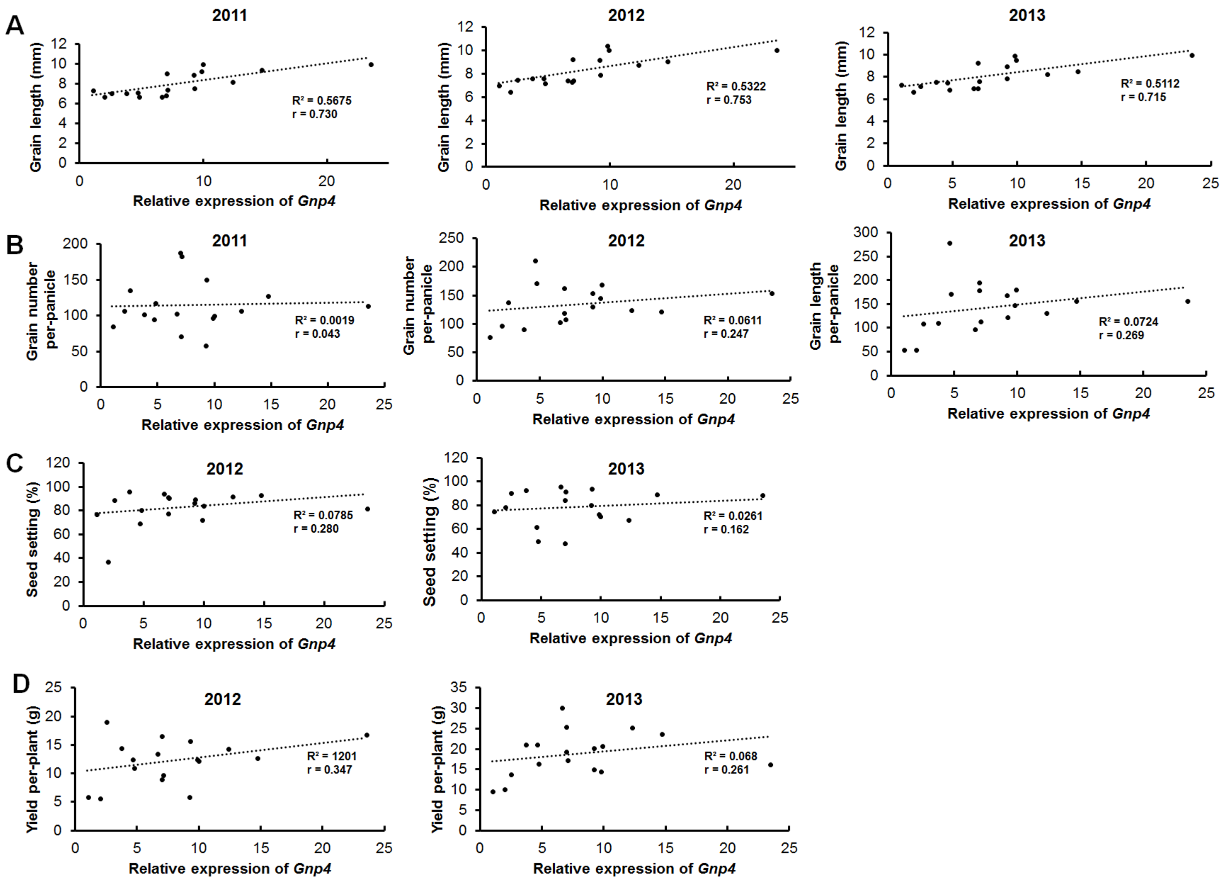

Figure S1. Analysis of the correlation between *Gnp4/LAX2* mRNA levels and several agronomic traits in 17 *japonica* accessions.

Correlation between *Gnp4* expression levels and grain length (A), grain number per-panicle (B), seed setting (C) and yield per plant (D) in 17 *japonica* accessions selected from the rice mini core collection in two or three years.

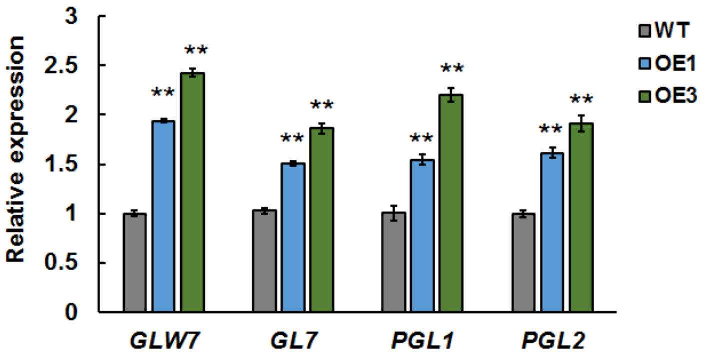

Figure S2. Relative expression levels of several genes related to grain length in rice.

Young panicles at 5 cm in length of Gnp4 overexpression and wild type (Nip) plants were sampled for analysis. Data are means ± s.e.m. (n = 3). Student's *t*-test. ** $p < 0.01$.

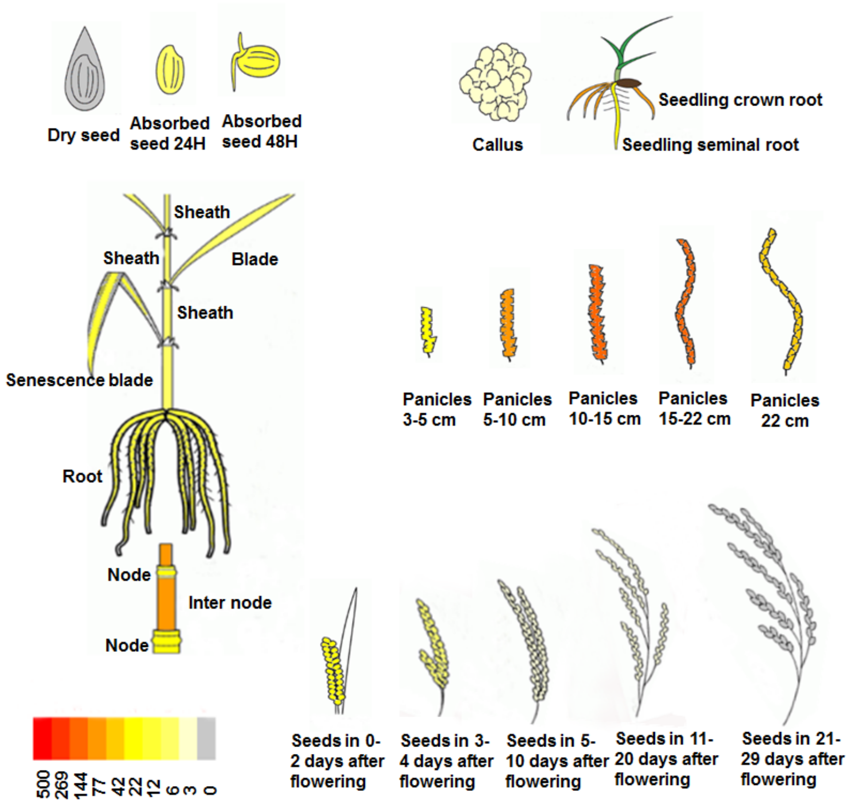

Figure S3. In silico expression analysis of *Gnp4/LAX2*.

In silico expression analysis of *Gnp4* carried out using HanaDB-OS (http://evolver.psc.riken.jp/seiken/OS/index.html; data downloaded June 2016)

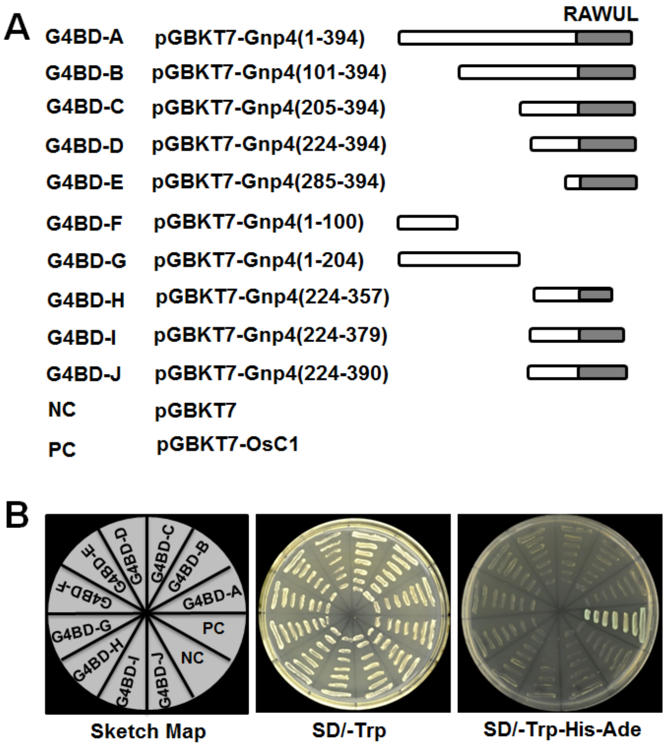

Figure S4. Auto-transcriptional activation activity analysis of Gnp4/LAX2 in yeast cells. (A) Schematics of full length and truncated Gnp4 baits. The RAWUL domain at the C terminus of Gnp4 is indicated with gray boxes. (B) No transcriptional activation activity of entire or truncated Gnp4 was observed. OsC1 was used as a positive control.

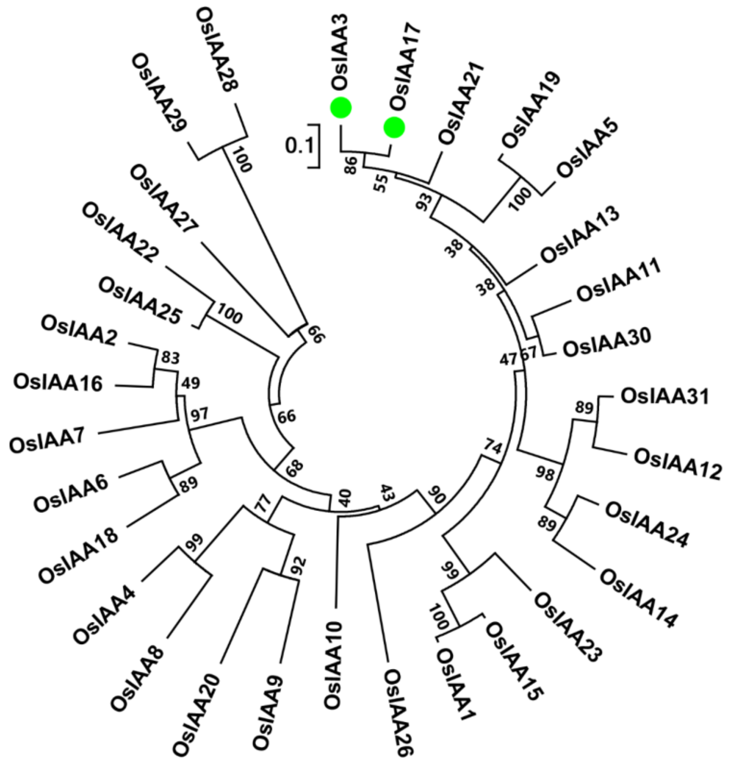

Figure S5. Phylogenetic tree of Aux/IAA proteins in rice.

The tree was generated using MEGA6.0 program by neighbor-joining method. OsIAA3 and OsIAA17 clustered on the same branch were indicated with green circles.

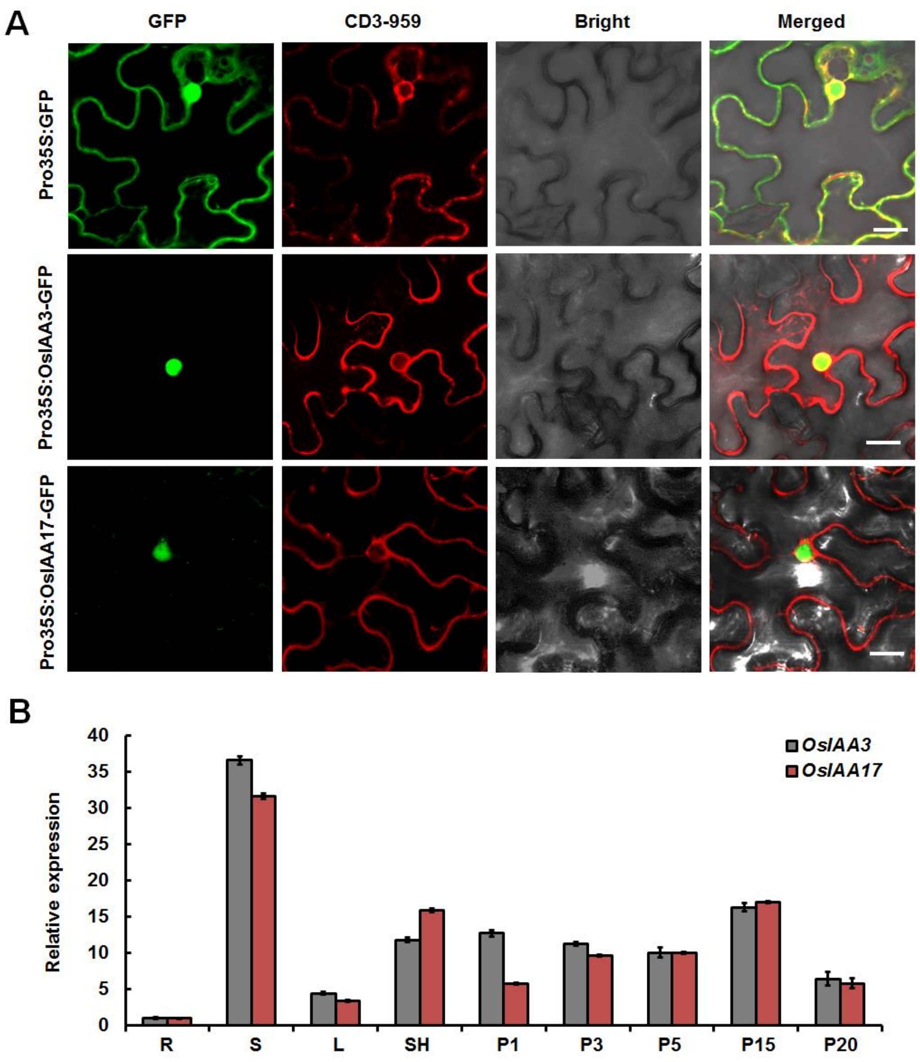

Figure S6. Subcellular localization and expression pattern of OsIAA3 and OsIAA17.

(A) Subcellular location of Pro35S:Gnp4-OsIAA3 and Pro35S:Gnp4-OsIAA17 in tobacco epidermal leaf cells. CD3-959, is a mCherry marker (*ER-rk CD3-959*). Scale bar, 20 µm. (B) qRT-PCR analysis of relative expression levels of *OsIAA3 and OsIAA17* in different tissues from Nip. R, root; S, stem; L, leaf; SH, leaf sheath; P, panicles, with the following number indicating its length (cm). Data are means ± s.e.m. (n = 3).

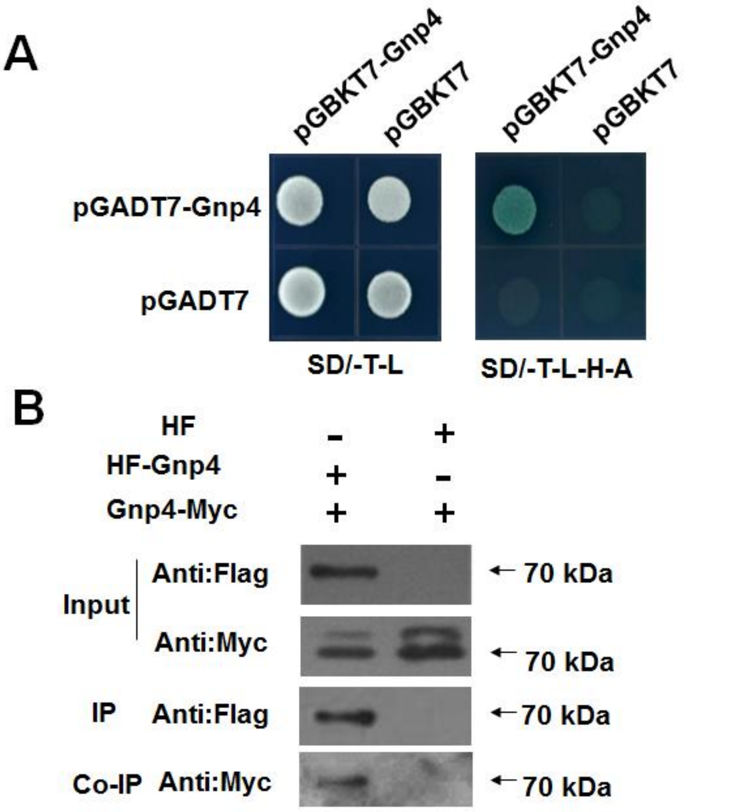

Figure S7. Gnp4 forms a dimmer in yeast and plant cells.

(A) Yeast two-hybrid assays showing that Gnp4 interacts with itself *in vitro*. SD/-T-L, selective medium lacking Trp and Leu. SD/-T-L-H-A, selective medium lacking Trp, Leu, His and Ade. (B) Co-ip assays showing that Gnp4-Myc interacted with HF-Gnp4 *in vivo*. Gnp4-Myc fused protein can be co-immunoprecipitated by anti-Flag beads when co-expressed with HF-Gnp4, but not the HF alone. The blots were probed by anti-Myc or anti-Flag.

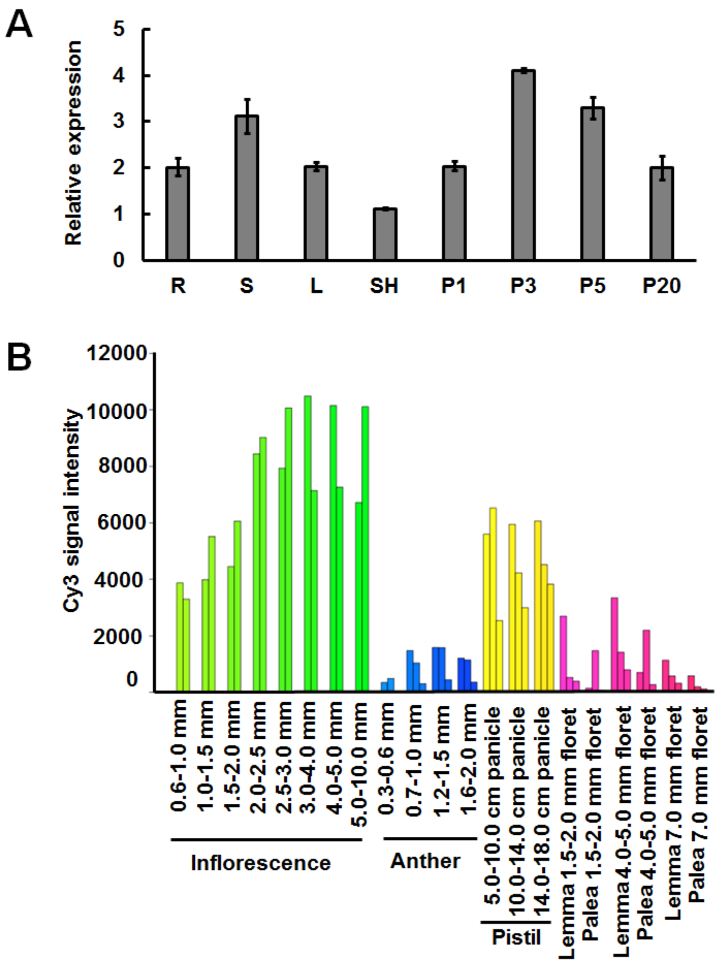

Figure S8. Expression pattern analysis of *OsARF25*

(a) qRT-PCR analysis of relative expression levels of *OsARF25* in different tissues from Nip. R, root; S, stem; L, leaf; SH, leaf sheath; P, panicles, with the following number indicating its length (cm). Data are means ± s.e.m. (n = 3). (b) Expression patterns of *OsARF25* obtained from RiceXpro (http://ricexpro.dna.affrc.go.jp/index.html).

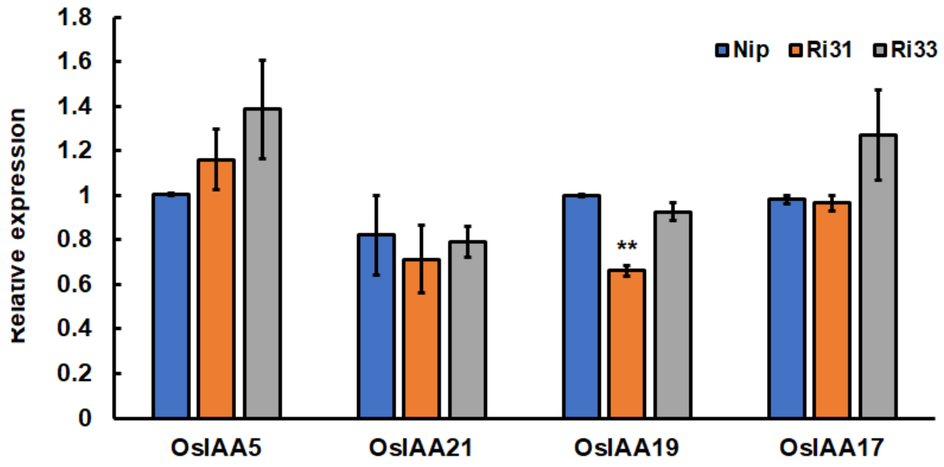

Figure S9. Relative expression levels of nearest homologous genes of OsIAA3 in wild-type Nipponbare and OsIAA3- RNAi plants.

Data are means ± s.e.m. (n = 3). Student's *t*-test. ** $p < 0.01$.

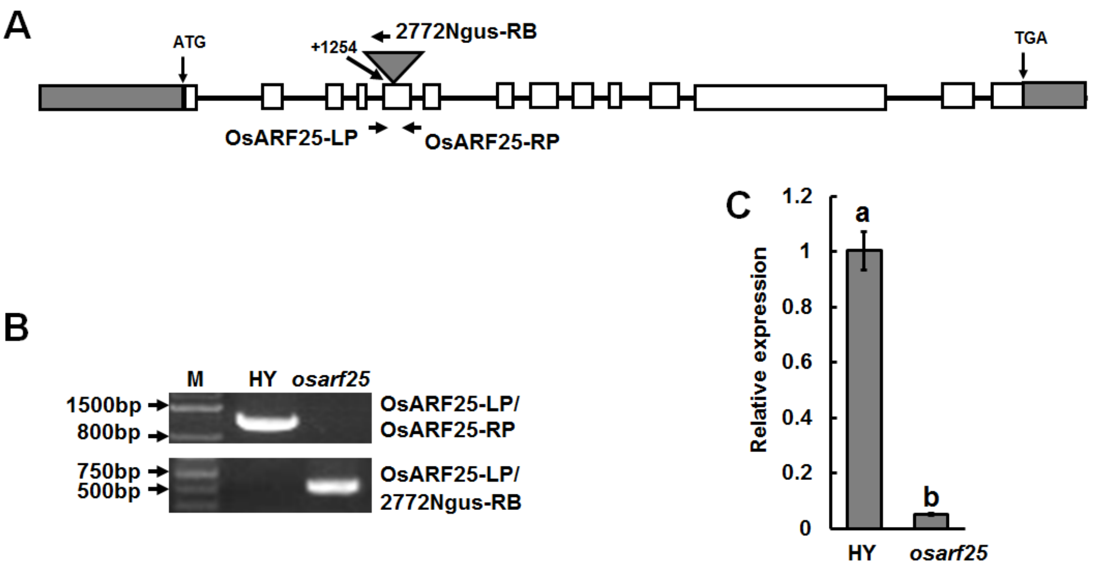

Figure S10. Identification of *osarf25*.

(A) Schematic of OsARF25; gray boxes indicate 5' and 3' UTR; white boxes are exons, and horizontal line represent introns. The T-DNA insertion is marked with a triangle and arrows indicate the primers used for genotyping. (B) PCR identification of *osarf25* at the genomic DNA levels. (C) qRT-PCR analysis of relative expression of OsARF25 in osarf25 and wild type plants (HY). Data are means ± s.e.m. (n = 3 plants with each 3 technical repeats, the presence of the same lowercase letter above the error bar denotes a non-significant difference between the means, P>0.05, Student's *t*-test).

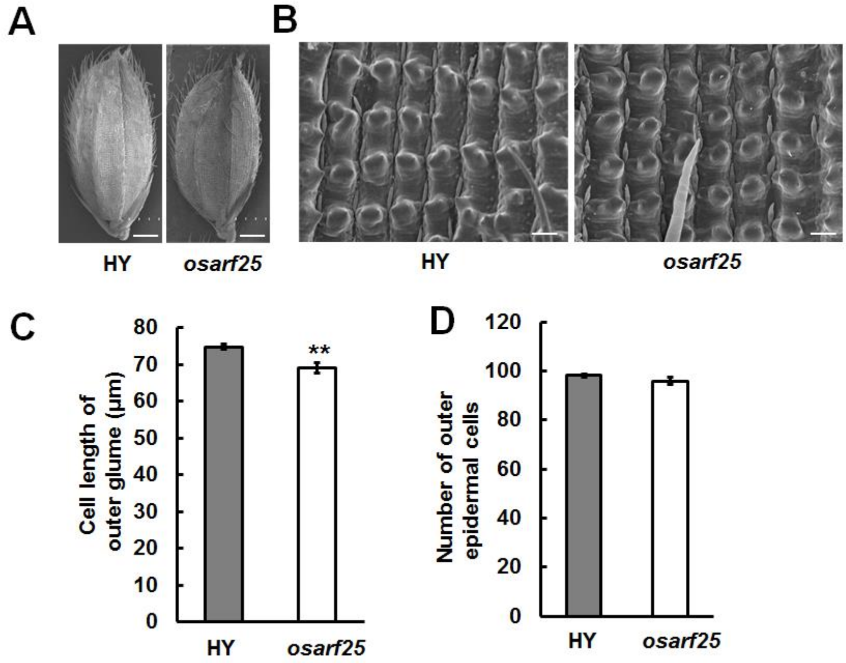

Figure S11. Scanning electron microscopy of glumes of wild-type Hwayoung and *osarf25*. (a-b) Morphologies of entire grains under SEM (a), and enlarged images of outer surfaces of glumes (b). Scale bars, 1 mm in (A) and 50 μm in (b). (c-d) Cell length (c) and numbers (d) in outer glumes of HY and *osarf25*. Data are means ± s.e.m. (n = 12 grains). P value was calculated by student's *t*-test. ** p <0.01.

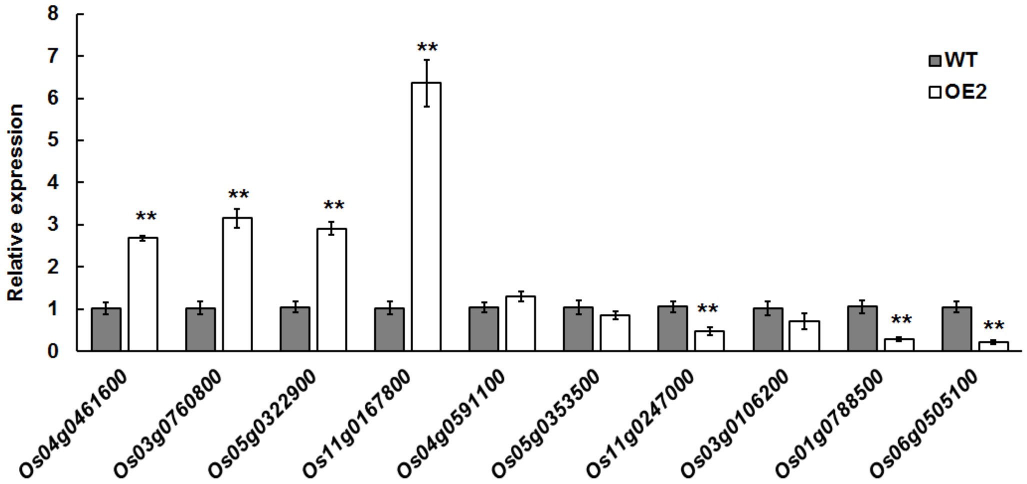

Figure S12. Validation of transcriptome data by qRT-PCR.

Five up-regulated genes and five down-regulated genes were randomly selected and subjected to analysis. Data are means ± s.e.m. (n = 3). P values were calculated by student's *t*-test. ** p <0.01.

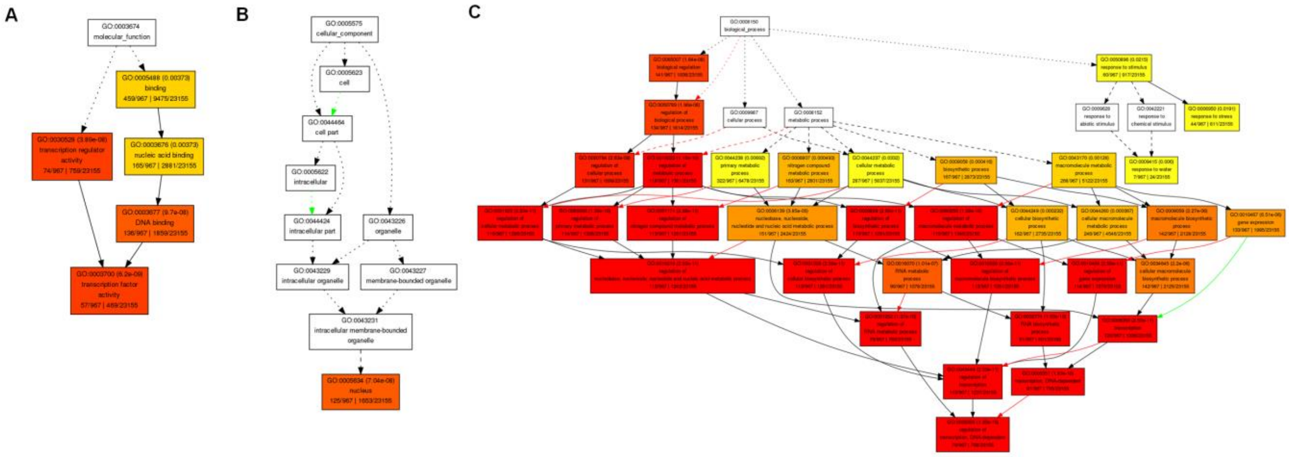

Figure S13. Gene ontology analysis of DEGs.

Information within boxes for significant GO terms include: GO term, adjusted p-value, GO term description, item number mapping the GO in the query DEGs and background, and total number of query DEGs and background. Significant GO terms associated with molecular function are in (A), associated with cellular component are in (B), and those associated with biological process are in (C).

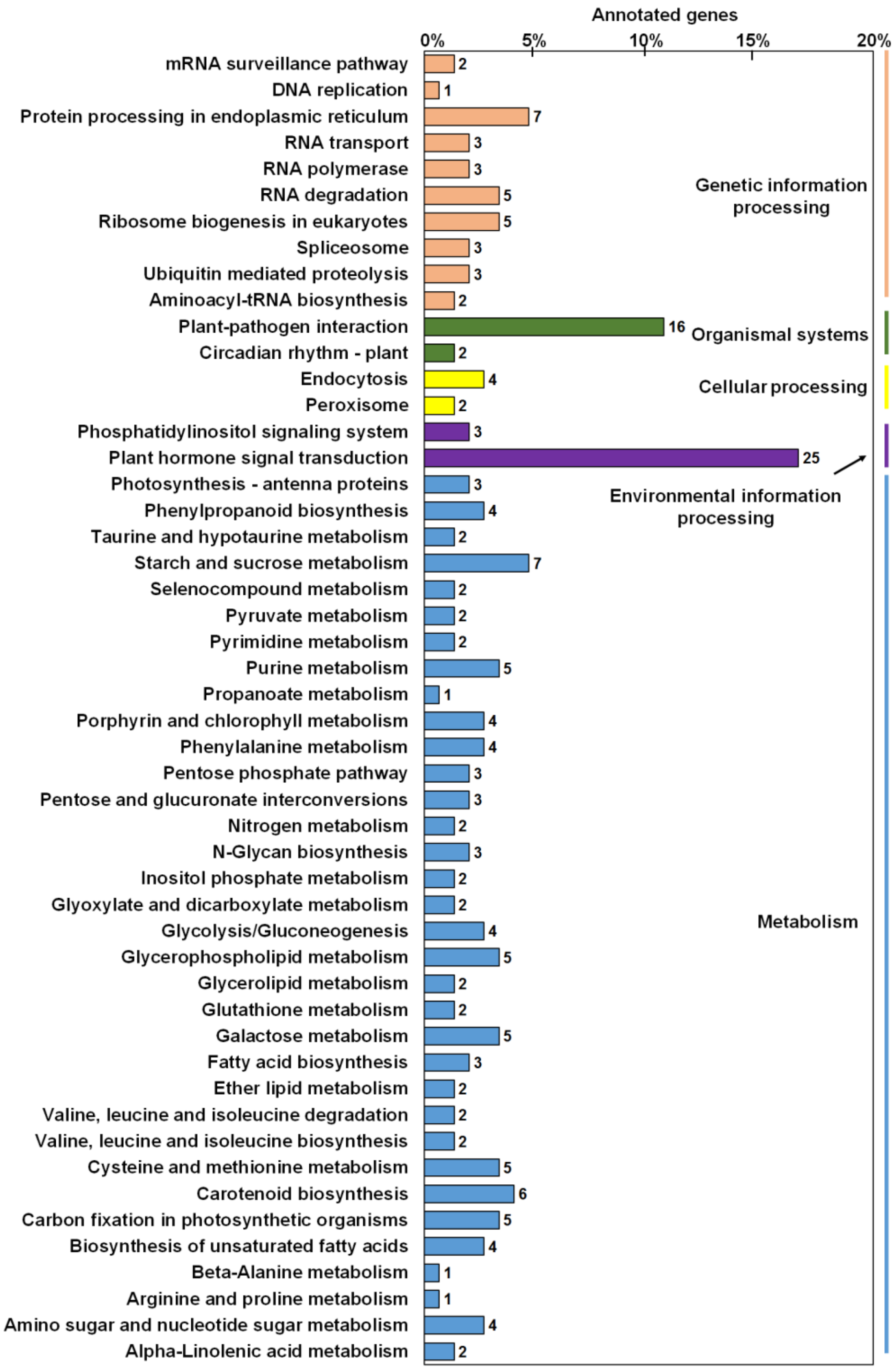

Figure S14. KEGG pathway analysis of DEGs.

Pathways listed in the left column can be divided into five groups as listed in the right column. Number above the column represents the item number mapping the corresponding KEGG pathway.

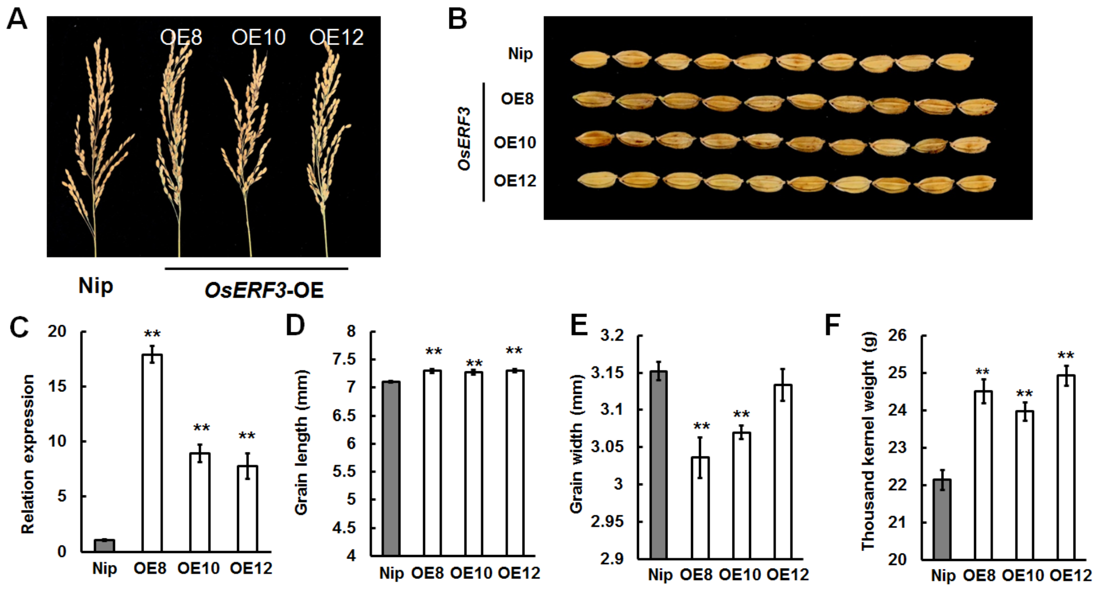

Figure S15. Phenotypic analysis of OsERF3-overexpression plants and wild-type plants. (A-B) Phenotypes of panicles and grains from *OsERF3* overexpression and wild type plants. (C) Relative expression levels of *OsERF3* analyzed by qRT-PCR. Data are means ± s.e.m. (n = 3). (D-E) Statistical results for grain length (D), grain width (E) and thousand kernel weight (F) of Nip and *OsERF3* overexpression plants. Data are means ± s.e.m. (n = 10). All p values were calculated by student's *t*-test. ** p <0.01.

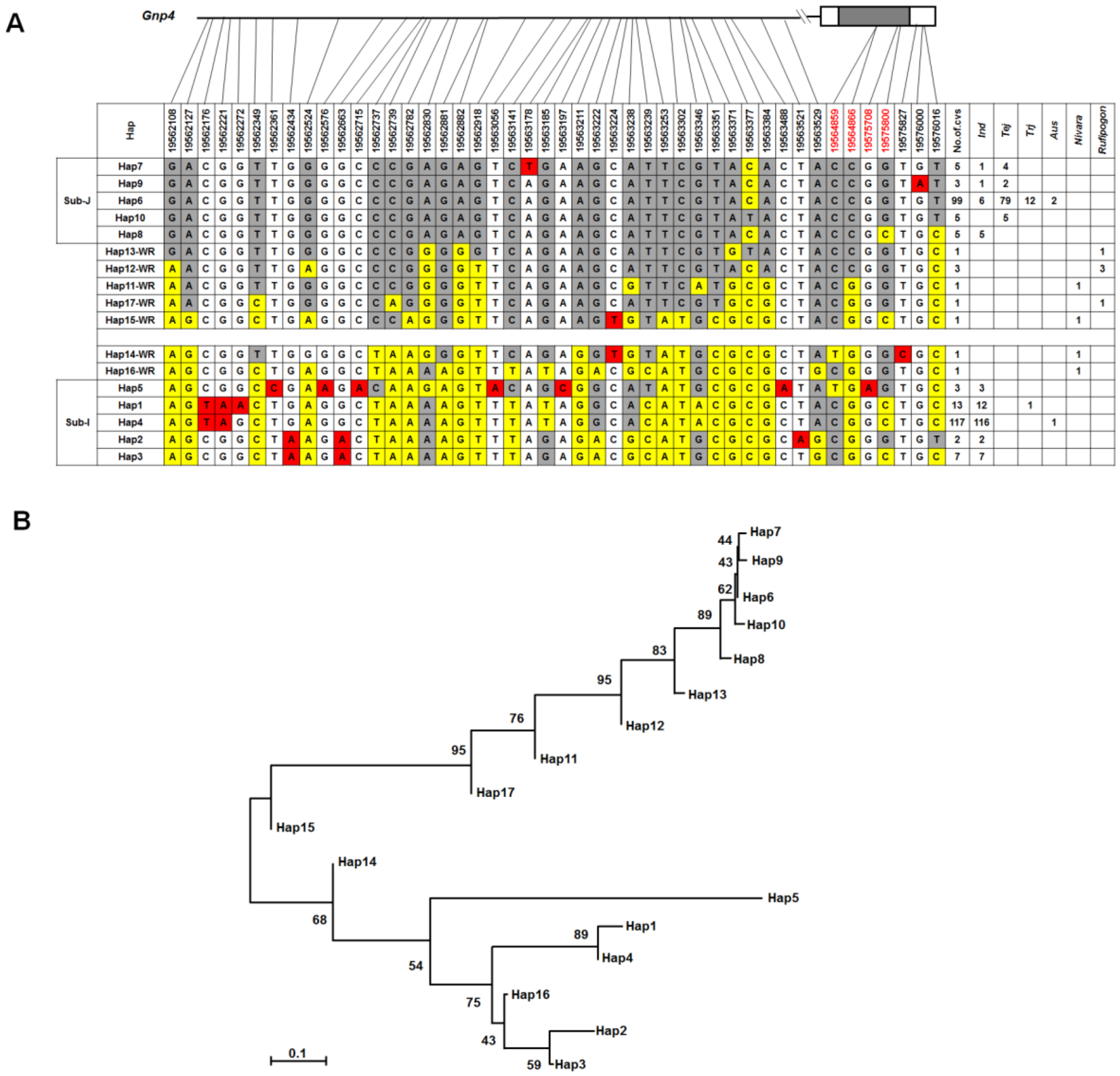

Figure S16. Haplotype Analysis of *Gnp4/LAX2*

(**A**) Haplotypes of *Gnp4* in 259 cultivated and 9 wild rice accessions. The position of each SNP is shown in the first row. Seventeen haplotypes were classified into two groups sub-I and sub-J. The number of cultivars (No. of cvs.) in each haplotype is listed in columns on the right. Grids in yellow background indicate the *indica* haplogroup; those in grey are *japonica*, and red indicates the non-typical novel mutations. WR, wild rice; *Ind*, *indica*; *Tej*, temperate *japonica*; *Trj,* tropical *japonica.* The SNPs locations present in the coding sequence of *Gnp4* are marked in red. (**B**) Phylogenetic tree of 17 *Gnp4* haplotypes. Scale bar indicates the average number of substitutions per site in different haplotypes.
