# Supplementary material for: Gnp4/LAX2, a RAWUL protein, interferes with the OsIAA3–OsARF25 interaction to regulate grain length via the auxin signaling pathway in rice
Source: J Exp Bot. 2018 Jul 28;69(20):4723–37. doi: 10.1093/jxb/ery256 (PMC6137978; doi:10.1093/jxb/ery256)
Supplement: Supplementary Tables S1-S5 [file ery256_suppl_supplementary-tables-s1-s5.pdf]

Supplemental tables

Table S1. Gnp4/LAX2- interacting proteins isolated by yeast two-hybrid assays

ID	MSU_ID	Annotation
GIP1	LOC_Os06g44450	CCT/B-box zinc finger protein, putative, expressed
GIP2	LOC_Os06g06530	proline-rich cell wall protein-like, putative, expressed
GIP3	LOC_Os07g43720	DCN1, putative, expressed
GIP4	LOC_Os05g34070	DIP1, putative, expressed
GIP5	LOC_Os05g28980	drought induced 19 protein, putative, expressed
GIP6	LOC_Os01g48190	drought induced 19 protein, putative, expressed
GIP7	LOC_Os05g02070	expressed protein
GIP8	LOC_Os02g05710	expressed protein
GIP9	LOC_Os03g58070	frigida, putative, expressed
GIP10	LOC_Os08g42740	IBR domain containing protein, putative, expressed
GIP11	LOC_Os02g38050	joka2, putative, expressed
GIP12	LOC_Os03g07880	nuclear transcription factor Y subunit, putative, expressed
GIP13	LOC_Os03g22740	nucleolar protein NOP5-1, putative, expressed
GIP14	LOC_Os05g14180	OsIAA17 - Auxin-responsive Aux/IAA gene family member, expressed
GIP15	LOC_Os01g13030	OsIAA3 - Auxin-responsive Aux/IAA gene family member, expressed
GIP16	LOC_Os04g52050	TNP1, putative, expressed
GIP17	LOC_Os10g42410	zinc-binding protein, putative, expressed
GIP18	LOC_Os06g47890	OsFBO9 - F-box and other domain containing protein, expressed
GIP19	LOC_Os01g17390	OsFBX5 - F-box domain containing protein, expressed
GIP20	LOC_Os02g36974	14-3-3 protein, putative, expressed
GIP21	LOC_Os03g28940	OsJAZ6, ZIM domain containing protein, putative, expressed
GIP22	LOC_Os04g02820	elongation factor, putative, expressed
GIP23	LOC_Os02g37290	Heavy metal transport protein

Table S2. Enriched GO terms in significant DEGs.

GO term	Ontology	Description	Number in input list	Number in BG/Ref	p-value	FDR
GO:0006350	P	transcription	120	1336	3.1E-14	2.5E-11
GO:0031323	P	regulation of cellular metabolic process	116	1295	1E-13	2.5E-11
GO:0045449	P	regulation of transcription	113	1237	6.8E-14	2.5E-11
GO:0019219	P	regulation of nucleobase, nucleoside, nucleotide and nucleic acid metabolic process	113	1243	9.1E-14	2.5E-11
GO:0009889	P	regulation of biosynthetic process	113	1261	2.2E-13	2.6E-11
GO:0010556	P	regulation of macromolecule biosynthetic process	113	1261	2.2E-13	2.6E-11
GO:0031326	P	regulation of cellular biosynthetic process	113	1261	2.2E-13	2.6E-11
GO:0010468	P	regulation of gene expression	114	1279	2.4E-13	2.6E-11
GO:0051171	P	regulation of nitrogen compound metabolic process	113	1261	2.2E-13	2.6E-11
GO:0019222	P	regulation of metabolic process	118	1381	1.2E-12	1.1E-10
GO:0006355	P	regulation of transcription, DNA-dependent	79	758	1.5E-12	1.3E-10
GO:0051252	P	regulation of RNA metabolic process	79	760	1.7E-12	1.4E-10
GO:0080090	P	regulation of primary metabolic process	114	1326	2.1E-12	1.6E-10
GO:0060255	P	regulation of macromolecule metabolic process	115	1345	2.3E-12	1.6E-10
GO:0006351	P	transcription, DNA-dependent	81	799	2.8E-12	1.8E-10
GO:0032774	P	RNA biosynthetic process	81	801	3.2E-12	1.9E-10
GO:0050794	P	regulation of cellular process	131	1699	4.6E-11	2.6E-09
GO:0065007	P	biological regulation	141	1936	3E-10	0.000000016
GO:0050789	P	regulation of biological process	134	1814	3.8E-10	0.00000002
GO:0016070	P	RNA metabolic process	90	1078	2.1E-09	0.0000001
GO:0034645	P	cellular macromolecule biosynthetic process	142	2125	0.000000047	0.0000022
GO:0009059	P	macromolecule biosynthetic process	142	2128	0.000000051	0.0000023
GO:0010467	P	gene expression	133	1995	0.00000015	0.0000065
GO:0006139	P	nucleobase, nucleoside, nucleotide and nucleic acid metabolic process	151	2424	0.00000094	0.000038
GO:0044249	P	cellular biosynthetic process	162	2735	0.0000059	0.00023
GO:0044260	P	cellular macromolecule metabolic process	246	4546	0.0000098	0.00037
GO:0009058	P	biosynthetic process	167	2873	0.000011	0.00042
GO:0006807	P	nitrogen compound metabolic process	163	2801	0.000014	0.00049
GO:0043170	P	macromolecule metabolic process	268	5122	0.000037	0.0013
GO:0009415	P	response to water	7	24	0.00018	0.006
GO:0044238	P	primary metabolic process	322	6478	0.00022	0.0069
GO:0006950	P	response to stress	44	611	0.00063	0.019
GO:0050896	P	response to stimulus	60	917	0.00072	0.021
GO:0044237	P	cellular metabolic process	287	5837	0.0012	0.033
GO:0003700	F	transcription factor activity	57	469	1.1E-11	6.2E-09
GO:0030528	F	transcription regulator activity	74	759	1.3E-10	0.000000039
GO:0003677	F	DNA binding	136	1859	5E-10	0.000000097
GO:0005488	F	binding	459	9475	0.000032	0.0037
GO:0003676	F	nucleic acid binding	165	2881	0.000028	0.0037
GO:0005634	C	nucleus	125	1653	4.2E-10	0.00000007

Table S3. Significantly enriched protein domains of up- and down-regulated genes in *Gnp4/LAX2*-overexpression compared to wild-type plants.

Category	Term	Count	Percentage(%)	P-Value	Genes	List Total	Pop Hits	Pop Total	Fold Enrichment	Bonferroni	Benjamini	FDR
	SM00380:AP2	26	3.576341128	5.14E-14	OS09G0522100, OS02G0657000, OS05G0497300, OS01G0693400, OS02G0764700, OS01G0313300, OS06G0127100, OS04G0529100, OS03G0183300, OS07G0674800, OS04G0546800, OS04G0610400, OS01G0797600, OS02G0654700, OS04G0550200, OS05G0389000, OS08G0474000, OS05G0361700, OS09G0457900, OS05G0497200, OS05G0473300, OS04G0399800, OS03G0191900, OS02G0677300, OS04G0669200, OS09G0522000	221	109	6051	6.531	0.000	0.000	0.000
	SM00774:WRKY	12	1.650618982	0.0000428	OS06G0649000, OS01G0750100, OS02G0265200, OS01G0821600, OS04G0287400, OS05G0343400, OS02G0462800, OS01G0826400, OS09G0334500, OS01G0246700, OS01G0624700, OS05G0322900	221	71	6051	4.628	0.003	0.002	0.045
Up-regulated	SM00054:EFh	15	2.063273728	0.000102	OS04G0492800, OS01G0135700, OS01G0955100, OS05G0223000, OS06G0172200, OS03G0743500, OS08G0540400, OS01G0949500, OS07G0681400, OS03G0331700, OS05G0380900, OS09G0412300, OS05G0585500, OS02G0126400, OS04G0584600	221	120	6051	3.423	0.007	0.002	0.106
	SM00331:PP2C_SIG	11	1.5130674	0.000474	OS03G0207400, OS05G0457200, OS09G0325700, OS01G0656200, OS01G0295700, OS01G0846300, OS03G0292100, OS03G0821300, OS03G0268600, OS03G0761100, OS01G0583100	221	78	6051	3.861	0.034	0.009	0.493
	SM00332:PP2Cc	11	1.5130674	0.000474	OS03G0207400, OS05G0457200, OS09G0325700, OS01G0656200, OS01G0295700, OS01G0846300, OS03G0292100, OS03G0821300, OS03G0268600, OS03G0761100, OS01G0583100	221	78	6051	3.861	0.034	0.009	0.493
Down-regulated	SM00028:TPR	10	2.83286119	0.000296687	OS04G0352400, OS07G0455100, OS08G0130300, OS05G0374500, OS01G0273500, OS03G0680200, OS03G0308800, OS01G0616300, OS02G0717400, OS04G0645100	124	107	6051	4.561	0.023	0.023	0.315

Table S4. DEGs containing AP2 domains in *Gnp4/LAX2*-overexpression compared to wild-type plants.

Gene-ID	Gene Name	FDR	log2FC	AuxRE(TGTCTC) sites
Os01g0313300	*OsERF68*	0.000000727	1.704051459	-1560~-1555
Os01g0693400	AP2/EREBP127	0.0000394	1.291098707	
Os01g0797600	*OsERF3/OsAP2-37*	0.00000148	1.304742366	-106~-101，-407~-402
Os02g0638650	*OsERF141*	0	2.450901144	-454~-449
Os02g0654700	*OsERF91/OsBIERF3*	0.001397606	2.235439953	
Os02g0657000	*OsERF35/ARAG1*	0.00346141	1.390733782	-1924~-1919，-682~-677
Os02g0676800	*OsERF20*	0	3.581204701	
Os02g0677300	*OsERF25/OsDREB1G*	0	3.15988512	-1842~-1837，-333~-328
Os02g0764700	*OsERF103/OsDERF5*	0	3.559439681	-680~-675
Os03g0183300	*OsERF64*	0.000221008	1.103327127	-1436~-1431，-514~-509
Os03g0191900	*OsERF47*	0	2.623410377	
Os04g0399800	*OsERF82*	0.000209479	2.386204889	-1988~-1983
Os04g0529100	*OsERF45*	0.006357848	1.387944484	
Os04g0546800	*OsERF1*	0.00000427	1.56306824	-1977~-1972，-1652~-1647，-1172~-1167
Os04g0550200	*OsERF34*	2.68E-12	1.837179625	
Os04g0610400	*OsERF77/OsAP2-39*	8.88E-16	1.946749093	-782~-777
Os04g0669200	*OsERF76*	4.37E-08	1.520373884	-1071~-1066
Os05g0361700	*OsERF61*	0.000000418	1.373219003	
Os05g0389000	*OsERF142*	0.000603571	2.420469575	-1187~-1182，-442~-437，-372~-367，-272~-267
Os05g0473300	*OsERF43*	0.0000659	3.357931515	-33~-28，-1991~-1986，-836~-831
Os05g0497200	*OsERF130/MFS1*	0.0000018	1.367061989	
Os05g0497300	*OsERF74*	2.74E-11	1.648698845	-1518~-1513
Os06g0127100	OsERF26/DREB1C/CBF1	0	4.932272731	-217~-222
Os07g0674800	*OsERF67*	0	3.599954454	-1633~-1628
Os08g0474000	OsERF104/DERF3	0	5.120850387	
Os09g0457900	*OsERF102/OsEATB*	0	4.241677944	
Os09g0522000	*OsERF31/OsDREB1B*	0.004577427	2.554335891	-1315~-1310
Os09g0522100	*OsERF133/OsDREB1H*	0.00000215	2.786528783	-1478~-1473

Table S5. The nucleotide diversity of *Gnp4/LAX2*

Taxon	N	S	H	π_T	Tajima's *D*	Fu Li's *D*
Wild rice	9	434	9	0.0021	0.16657	0.16945
O.sativa	249	665	205	0.00211	1.6408	-0.94294
ssp. japonica	95	367	54	0.00016	-2.82607***	-8.95937**
ssp. indica	154	630	154	0.00093	-1.15007	-0.37515

N, total number of accession sequences; S, number of polymorphic (segregating) sites; H, number of haplotypes; π_T, average number of nucleotide differences per site calculated based on the total number of polymorphic sites; ***P* <0.01, ****P* <0.001.
