# Supplementary material for: Gnp4/LAX2, a RAWUL protein, interferes with the OsIAA3–OsARF25 interaction to regulate grain length via the auxin signaling pathway in rice
Source: J Exp Bot. 2018 Jul 28;69(20):4723–37. doi: 10.1093/jxb/ery256 (PMC6137978; doi:10.1093/jxb/ery256)
Supplement: Supplementary Dataset S1 [file ery256_suppl_supplementary_dataset-s1.pdf]

```
Majority        XXXXXXXXXXXXXXXXXXXXXXXXXXXXXXXXXXXXXXXXXXXXXXXXXXXXXXXXXXXXXXXXXXXXXXXXXXXXXXXXX
                         10        20        30        40        50        60        70        80
Gnp4                                                                                              81
Sb06g014520                                                                                       81
XP_003579650.1                                                                                    81
XP_008663842.1                                                                                    81
Os12g0600200                                                                                      81
Os03g0632800    MQPAPASPPKADGGEDEEEECSRAVVKEEPHHQQEEDDDDAAAAADGGEDEKEKVEEEEVEERGRRRRGRPGRKRGRRSGG 81
Os03g0798200                                                                                MQGAT 81
Os03g0741700                                                                                      81
Os12g0479100                                                                                      81
Os01g0928400                                                                                      81
At1g06770                                                                                         81
At3g23060                                                                                         81
At2g30580                                                                                         81
At1g28080                                                                                         81
At5g13250                                                                                         81

Majority        XXXXXXXXXXXXXXXXXXXXXXXXX-----XCXXLLRDATTISECLH-TFCRXCIXEKITDEEVESCPVCNIDLGHRPLEK
                       90        100       110       120       130       140       150       160
Gnp4                          MVPARSLAHPH-----PHLVRRRRDHAAAAHGATARCDDDDDGVVTPRGPTRYMAQEPINHHQHQH- 156
Sb06g014520                   MVPARN     -----LQPPLLRDAHATPCAAAAAAG     LGVTPTAASSHMAQEPIDKHHSEPS 157
XP_003579650.1                MVPAR      ----- NLQLLRDEHGTPPFVAR        GVDAVTAQPHHMAQEPINQESPKP- 156
XP_008663842.1                           -----                                     MAQDPSRPHRHSSK 157
Os12g0600200                             MKYAPIY      LLTLS-CLHYAVCRKCIYKKINDEELEHCPVCKIDLGCAPLEK 161
Os03g0632800    GGGSAAAAAAARGGVVMVKRELLARCMTCPLCGRLLRDATTVSECLH-TFCRKCIYEKLNDEEVESCPVCKIDLGCTPVEK 161
Os03g0798200    PGDAAGEEVGGGGDVVMVRRASVAACLTCPLCGRLLRDATTISECLH-TFCRKCIHEEFVDKESCCCPTCNIDLGCAPLEK 161
Os03g0741700           MGAARGGVRRVRREALVACMTCPLCKGLLREATAITECLH-TFCKECIMEKIDDEEVDHCPVCNIDLGCDPEEK 161
Os12g0479100                             -----                                     MAQDPSHPHRQS K 157
Os01g0928400                             -----                            MVPARSRLFHHDSSSSSKRGGGN 157
At1g06770                    MMIKVKKETMRACLSCSICDNILRDATTISECLH-TFCRKCIYEKITEDEIETCPVCNIDLGSTPLEK 161
At3g23060                    MLTKVLSKEVKPCLACPICTNPFKDATTISECLH-TFCRSCIRNKFINERVNACPVCNVNLGVFPLEK 161
At2g30580                MEGDMVAKVKRETVVACMTCPLCDKLLRDATTISECLH-TFCRKCIYEKITEDEIESCPVCDIDLGGTPLEK 161
At1g28080                                -----             MTNMVPLESSTLFGVYGGFTDSMMMRETTTNNQDPSGD 157
At5g13250                                -----    MVSAAESSSS---------------TSTLLLPCAFANSFAFMRLLQK 142
```

```
Majority         LRPDHNPQDVRAKIFXXPLKRKKVQAPEVAAHVVLPAKRKERSXSSLVVXTPXVSAQAGTTGRRTKAXTRKXAGALXXXXX
                         170       180       190       200       210       220       230       240
Gnp4             -DPPKQPPPR---------EADD---DH---HRIQE  -----REPLPPPTTTTRNQRLQLQLGGDGHHN-----------   205
Sb06g014520      TKPPPPPPPQERQQQQQQA  DDQHTRHAHAHREQQQQLTLR   PLQPAGSSHQEAAGTSGSSSGG SSGNGGAG         238
XP_003579650.1   -QQ HQVPPQ---------EEEDEQP  ---       AVLP-PP                  QPAGTSGSSSG           223
XP_008663842.1   DTAAQQPEPEHQHEHEQQP  PAEILHQLAPQAQPQPPHDVH           EPAAAASSSSS SDAGS              238
Os12g0600200     LRADHNIQDVRSKIF  PLKRKKVNAEEVESPIAPPAKRKERSISSLVVNTPEITP KSLTGRRTRASTRKSAAALRDLGP    242
Os03g0632800     LRADHNLQDVRSKIF  PFKRKKISADEVAAPVLLPSKRKERSISSLVVDTPTVTP TGLTGRRTRAVTRK AAALR----    238
Os03g0798200     LRVDHSMQFVRSRIF  PFKRRKVENPEIICPVASPVKRKERSLSSLTIPAPQVSIQKCLTKRRTKASC------LR       236
Os03g0741700     LRPDHNVQDIRNKVF  PLKVKKVGAP KAPTVTLPVKRKQRSLSSLVVDTPRVAVQTGLTGRRTKTARRTAVSHVNSPGN  242
Os12g0479100     DTA----------------AAP--------------------PPPPPQEQPQQQQ--------  P    ELITAPPPR    193
Os01g0928400     SSSSCTPQYCIATQLDAAAAAAPARRRHHHHHQLAMAPPEDD           AAAGTSSRGGGAGDDDDGDK          238
At1g06770        LRPDHNLQDLRAKIF  ALKRRKVKAPGIVS   LPGKRKERSISSLVVSTPMVSAQAGTTRRRTKAPTRK   ELR NGS   242
At3g23060        LRSDCTWQDLKLKIYRAMMESLKKAGPKTVAASVKSSKKKRKSRTSLRVSSSRVSSSPDTPLEPANVVVEPPNVVVEEKHR  242
At2g30580        LRPDHILQDLRAKLF  PLKRKKERAPEVVSSISLPAKRKERSISSLVVSTPRVSAQAGTTGKRTKAA---TRK   D---   236
At1g28080        TCSVSDPAPGSTDYYNISSSPVKDDGTAPKNDVVCSINVDDNKDKSWLRLGIGPEENTDGSYKLQRCCSKNGSGRE      238
At5g13250        THHFSAVSEDDDNNKDIHLGSDHHQAKDNNHHQV------FRRECSFMADQEEPRSTTSRLSSKEDDHDNNS-----NNNN  212

Majority         XXXXXXXXXXXXXXXXXXXXXXXXXXXXXXXXXXXXXXXXXXXXXLXXXXXKXPQXXRXXXXXXXXXSXSXXEXXXXXXXX
                       250       260       270       280       290       300       310       320
Gnp4             -----------HHHHHHQEVAGTSGSSSGGSSSNNGGGG                                            275
Sb06g014520                                            DWLRLGLAPAYPSAA----------------------------  292
XP_003579650.1   GNSSSNGGGGG                                        GGGDWLRLGLGPSAAASASGTEADLFAAAD  304
XP_008663842.1                                         SWLQLGIGGPSPSSPP  RRKRPRRDDD                 319
Os12g0600200      IIPPVKKD            SDNTNKNADNSS------------LLDSLSKVPQTRRQVLSNAETSSHPSSKDKGGDDKD  311
Os03g0632800     G--------LGPGIDDPVKKE            IDNGEKHAQNSSLPTNLGKVPQTRRQMSSNAEASNHSSNKDTEGDRKD  311
Os03g0798200                                              NFPLHST------SR         GSKDTSKKLGG       311
Os03g0741700     NGTIKLANK            SEGRDHKTQKIS----------AAQSAKMTKTGNKKKNNTDVDVTIQSSSEDRKD  DHT  313
Os12g0479100     DDVAHQ             EPSTSSSSGGGGTDAAG                                               274
Os01g0928400                                           DWLQLGLAAAAPTATA----PAAAAAPSSSTPTAP          315
At1g06770        LAERTVKKE            ESSGDELLESTS------------SPDTLNKFTQNKRQSKKS CKESISNKENKDGD  E  311
At3g23060        ETVLAL---------QSTRKPIITFQKRGRKASLP---KKIDSKPEPELPPKEPKIKNLFDLNNEPEDNGLDEAEGSTFQE  311
At2g30580        ------VRGSGSFTKRTVKKE            EEFGDDHVESASSPETLKKFTQNKRQSSYANPNQSLSNRRNKDVD  E  311
At1g28080                                              NSLELSLFSSSSTAVS---------SSIDHLPTQPLQLPYHH   310
At5g13250        INNNNNNEAEDHEMRQQGWLR                                          LSLGHEEDVKPDLD      293
```

```
Majority        XXXXXXXL-WKPLNXLVXXXXXRKXXXSXXXXXXXXXXXXXX---XXXXXXXXXXXXXXXXXXXXXXXXXXXXXXXXXXXEXXX
                     330       340       350       360       370       380       390       400
Gnp4            TRDW-LRLATGPASPGASAGSDHDLFPSTTTTAPAPQPPTP---TPTPTPTPTPRHHHHDVLVLPG MP PPGSFLRPGP   352
Sb06g014520     ------------THPGVAFAADRAAGPPSPLLLSSSEPQRP---P PPPPLLSSQRPPG ----MG--FPPGPFLHQAA   352
XP_003579650.1      -            RGPRQEMLVLPALDMPMPP---GAFPRPG               ------------         369
XP_008663842.1                      ---------           ---                                DDDA   388
Os12g0600200    LDKS EL-WRPLNCLVEAASKTKSYRSSS AARGNQPT                     ESP SSANASRTKAREY      391
Os03g0632800    LADKTDEL-WRPLNCLVE-----------AAN---------RTKSSRSSSQSPFVKREQLS                     371
Os03g0798200            -WRPLGCQLKLGKDKKSLKSSVKDTNRTKSKSG                  DTD DGAPASKAKAREP      391
Os03g0741700    I--DKEDL-KKPLNSLVDTANRTKFFRSGPKGQAAKEDKI----------------KNSI KLLA          EDDT   375
Os12g0479100       LSLQLGLGPSAPGSPSSSRRKR---------PRTDAHH---                                      343
Os01g0928400                                            ---                   ELDV-FAAKQY-----    387
At1g06770       PWDSKMD -WKPLN----------FLVEVANGTK-------PLKSSASQGSGSKSEHA                   NV   374
At3g23060       VVPKEKDL-CKPIFSLSVTLNINDTPPDIVEPEISSDDDTEESVEPIQNKCVVNRETKEVPVQVNQNSLLISSDRDREDNS 391
At2g30580       PWDSKLHL-WKPLNFLVDVANGTK          DPKSELG                  NASHNDVQGSKTKTKDH      391
At1g28080       DQLLTMR---GPS                           ---                  LVYNHQ-------        378
At5g13250       HRQ---QHQTDPT--------ARRDSFLELNLSSGGSNREEE---VGLPLSSLFHHQHQAGGMMINPL------------- 347

Majority        XXXXXX------XXXXXXXXXXXXXK---------GXXXX--XX-XXPXXLP-R--KRTGXXXRRP-------------
                    410       420       430       440       450       460       470       480
Gnp4            AMP                GIPQAS IPTHMLRAA   --  --PPW LPPWS-P VAAP   PPLLPFPHQ---HRA    425
Sb06g014520     HG                  IPQAS IALPVPRAG   --  --PPW LPPWS-PSAVAPPPPPSLIPFGF---GHGA    425
XP_003579650.1      IPQASMQIPDGGPRAG      PP---------WQMP--PW--SP A-------Q--QHQQQPFLLPFAHQAQAQRA 428
XP_008663842.1  G      PSTATSSVQPAAAPPPQLPPQLLLSLQPAGRPS    --SSSSSAP---APAPARAV-----------------  447
Os12g0600200        L--------------LKSKVQDEK---------K--E     VPVATVPF-KRKGPGRGRKPAQP-------------  433
Os03g0632800     DSP GSTSVNKTKSREY      MQK---------SKIEDDKKD     VPLLKRKNQRTG   RRR--------------  429
Os03g0798200        FT----------------------R---------               YGRAAKRTG    RK--------------  429
Os03g0741700    EDKLVVTGR----KVMPC      SNK---------LKVKEENNRS   PSQSASSKDKTTSDYELRK--------------  429
Os12g0479100                                      EAAAG        PSTS        AADPQLPELRLSVLHPG      424
Os01g0928400    -------------------NGR     PPLFPLPILRSYHHHHHQYG-----------HGGRCPPPSFFPFAR-------  432
At1g06770       SRNQFQGS---KTKTKNK      KRK---------CKREDDKSNNGDPTTSETVTPKRMRTTQRKR--------------  429
At3g23060       GQKL----------KTNGAATSRSRKKK---------GKKPVEKSYSLRPRIG-------------RR--------------  427
At2g30580         --------------    KRKCKLE-------------------EEISNNGDPTTSETATLKRTRRTRRKR--------------  429
At1g28080       ---LMKPQTLLNRG    FSFPSSKPWIPQYMAP-----------FRPSSLSDRDVTNNNSLSRSCCV------------  433
At5g13250       ---MFHTR                    PQDMIGPWAAA--AFRTP    FVPQNLTQPSSSSSGSLMMPLMGQYFGRSS      423
```

```
Majority          -----SAXT-GDSSGXDAP-RVVLAPPXXXXXX--AAGVWFSLQASPDQEREXPFLPQIPRSYLRIKDGNXMTVSLLQKYL
                      490       500       510       520       530       540       550       560
Gnp4              FYAAPPTTTPPASSGFDAI-RVVLPPS  AVAA--AAGVWFVLQAAPLQGRE PFLPQIPRSYLRIKDGR VTVRLLTKYL  503
Sb06g014520       FY      TPGAGSGLDAI-RVVLPP      S--AAGVWFALQAAPHQGRE PFLPQIPRSYLRIKDGR VTVRLLIKYL  503
XP_003579650.1    FYAPGSTAS---ASGFDTI-RVVLPP     PV--PAGVWFVLQAAPHQGRE PFLPQIPRSYLRIKDGR MPIRLLIKYL  503
XP_008663842.1    ------APPP  P AHE------------AG-T------WFLLRAAPNQRREEPPLPHIPRSYLRVRDGRMMTVRVVMRYL  503
Os12g0600200      -----PAAAV    SS--HS--ASKHE  KL  LT--PVWFSLIASFDQKGA PPLPQIPTHYLRIKDDN MPASSIQKYI  503
Os03g0632800      -----ELHAQSDSK  --  PEAAATQNE  KK  FSSIWFSLVASFEQEGD PPLPQIPSHYLRIKDGN IPASSIQKYL  503
Os03g0798200      -----KLLMLKNKK    KRFKAKQPS  KKRR--FRALWFYLLAAFDQRGV PTLPQLPAKYLRIKDVD LPASIIQKYL  503
Os03g0741700      -----GQHA--DSQQGQI GSTRTGALHDG IT   RPVWFLLVPSPDQKQD PRLPQLPTYYVRIKDGS LQTSLIQRYI  503
Os12g0479100      --PGPS    SSAS   AVGAVVAAAPPP  P AHEAGTWFVLRAAQNQRRE PPLPQIPRSFLRVRDGR MTVRVVMRYL  503
Os01g0928400      ----------PLRSSGDLPGVMRVISPPRRAAAADAAGLWLTLQASPDQFRE PILPQIPKSYLRIKDSN MKVEVVMKYL  503
At1g06770         -----SATTLGDSRN--LPQPDESSAKQE  RR  NGPVWFSLVASNDQEGG TSLPQIPANFLRIRDGN TTVSFIQKYL  503
At3g23060         -----TVNPAAGTTTPEAPVSVEEEMKVE  EGRNNNPVWFSLKPSKTQNIE MLLPPITACCIRVKDSN MTVSYLKKYL  503
At2g30580         -----SS TFGDSRIPLLPGAASLKQE  RR  NGH--VWFSLVASSNQEGE ASLPQIPANYLRIRDGN IPVSFIQKYL  503
At1g28080         -----------DEGGAGPSSEFRVLDPPR    RPHSGLWFLLQASQFQERE PFLPQVNKSYLRIKDGR ITVRLLIKYL  503
At5g13250         FQPQLIGNDNPDGVAGPSS-SLRVIDPPR    RPHSGIWFLLQASQNQTVE PFLPQIPKSYLRIKDGK MTVRLLMKYL  503

Majority          MNKLGLESESEXVEITCRGXPVXPXLTLQNL-----LWLXXR--XXXXXPXRLPASVGSSAKDFVMVLXYGRX--XXXXXX
                    570       580       590       600       610       620       630       640
Gnp4              VNKLGLEDESE VEITCRGRQLLPILTLQHV--RDSIW-CRRD-A  VSPSAAPDIPTADHHQHIMVLQYGRR--P       578
Sb06g014520       AGKLGLEDESE VRFIR         ILHHNSN     -    - LL--P                       --       578
XP_003579650.1    VNKLGLEDESE VEITCRGRPLLPFMTLQHL--RDSIW-CQRD-A  VSPLVAPDMSTAN   HIMVLQYGRR--T       578
XP_008663842.1    VNKLGLDDDSQ LEITCRGQRLQPTMTLQQV--RDTIWRPAVP-  AEAAAVLPA-PGSRSTDHIMTLHYCRS--        578
Os12g0600200      MQKLSLPSETE VEISCCGQPVNPIQPLRNLIE---RWLR---FGP   ARTLQTVVGSSGGDYVMVISYGRPKAA       578
Os03g0632800      MQKLGLPNEAE VEINCCGQPVNPTQPLCNLVE---VWLRGRS   TQTT---QTMIGSPAKEFVMVLTYGRPKAITP    578
Os03g0798200      AQKLNLSSETE VEVLCGGKVVNQGMTLHDLAD---CWLEK---GP   KSRMRSSVGSPATGFMVTLFYRRPDVDVSSSP 578
Os03g0741700      MNKLDLASEDE VEITCHGEAISPSTTLQGLLE---LWLKSS---P   VEQVQASLGAQAKEFVMELGYRRPQRPPSS   578
Os12g0479100      VNKLSLEDDS                   QV--KG        -       VRPS-VTSWVTPHD        --       578
Os01g0928400      AGKLGLTQSH                 LQRAASSSLL---AGQ-K  CQRQHLVLITATKLQGRRRRRRRG  --       578
At1g06770         MRKLDLESENE IEIKCMGEAVIPTLTLYNLVD---LWLQKSS---N  HQRFAALVGSSAKDFTMVLVYARKLPECNM   578
At3g23060         MVKLGLESEDQ VEIWLRDEPVCSSLTLHNLVD---WWVQTT---PL  PERQSAMVGSSAAEFIMDLYYSFKSDASDSGS 578
At2g30580         MRKLDLKSEDE VEITCMGEPVIPTLQLHSLVD---LWLETTS---K  HQRVAASIGSSAKEFVMVLVYSRKLPECNN   578
At1g28080         MKKLELDSESE VNI                            -                             --       581
At5g13250         VNKLRLEHESQQVEIRCRGQELEPVLTLQHV--RDAIWRGSRD-TSSLSQNITL-LPNSSTSDHLMVLHYGRT--IS     578
```

Majority	X X X X X X X X X X X X	
	650 660	
Gnp4		590
Sb06g014520		590
XP_003579650.1		590
XP_008663842.1		590
Os12g0600200		590
Os03g0632800		590
Os03g0798200	A P P Q P D T E S C H S	590
Os03g0741700		590
Os12g0479100		590
Os01g0928400		590
At1g06770		590
At3g23060	A S E	590
At2g30580		590
At1g28080		590
At5g13250		590

Decoration 'Decoration #1': Shade (with solid black) residues that match the Consensus exactly.
